# A Three-Dimensional LiDAR Observability Framework for Pedestrian Representation: Sensor Placement and Multi-View Fusion on a Compact Autonomous Vehicle

**DOI:** 10.3390/s26092670

**Published:** 2026-04-25

**Authors:** Juan Diego Valladolid, Juan P. Ortiz, Franklin Castillo, José Vuelvas, Chuan Yu

**Affiliations:** 1 Department of Automotive Engineering, Universidad Politécnica Salesiana, Cuenca 010107, Ecuador; fcastillog2@est.ups.edu.ec; 2 Department of Mechatronics, Universidad Politécnica Salesiana, Cuenca 010107, Ecuador; jortizg@ups.edu.ec; 3 Department of Electronics Engineering, Pontificia Universidad Javeriana, Bogotá 110321, Colombia; vuelvasj@javeriana.edu.co; 4 HanKaiSi Intelligent Technology Co., Ltd. (PIX Moving), Guiyang 550016, China; angelo@pixmoving.com

**Keywords:** LiDAR sensing, autonomous vehicles, pedestrian observability, sensor fusion, point cloud, sensor placement, ROS 2, experimental evaluation, 3D perception

## Abstract

Reliable pedestrian perception in autonomous driving depends not only on detecting the target, but also on how completely and consistently its three-dimensional geometry is captured from different sensor viewpoints. This study presents a LiDAR-based observability framework for evaluating pedestrian representation on the ANTA compact autonomous vehicle platform using a roof-mounted Top LiDAR (TL), a Front-Right LiDAR (FRL), and their fused configuration. The pedestrian was analyzed in a canonical local frame using geometric extent ratios, projected surface occupancy, voxel-based volumetric occupancy, and statistical descriptors of the local point distribution, integrated into a global observability score, $S_{3D}$. A Distance-Robustness Index (DRI), an overlap-based complementarity analysis, and a lightweight temporal centroid-sensitivity check over 20 consecutive frames were used to characterize performance across distance. Using ROS 2 bag data processed offline in MATLAB R2025b the fused configuration achieved the highest mean global score (0.563), compared with 0.504 for FRL and 0.432 for TL, and the highest robustness (DRI=0.5628, CV=10.7%). The results show that 1 m maximizes local density, 2–3 m maximize projected and volumetric completeness, and 7 m provides the best balanced observability. Within the evaluated platform and under the controlled benchmark conditions, complementary multi-LiDAR fusion provided the strongest overall geometry-aware pedestrian representation.

## 1. Introduction

Reliable pedestrian perception remains one of the most critical requirements in autonomous driving, particularly in urban and campus-scale environments where vulnerable road users frequently interact with vehicles at short and medium distances. In such scenarios, perception performance depends not only on whether a pedestrian is detected, but also on how completely and consistently the sensing system captures the target geometry. A more informative three-dimensional representation can directly benefit downstream tasks such as detection, tracking, motion interpretation, and risk assessment [1,2,3].

LiDAR sensors have become a central component of autonomous-vehicle perception because they provide direct range measurements, geometric structure, and relative robustness to illumination changes. Accordingly, a substantial body of recent research has focused on LiDAR-based object detection, semantic understanding, and multimodal fusion [1,2,4,5]. However, comparatively less attention has been paid to a more fundamental question that precedes many downstream perception tasks: how the mounting position of a LiDAR sensor affects the three-dimensional observability of a pedestrian, and to what extent complementary viewpoints improve the quality of the sensed representation [6,7,8]. This gap is reinforced by the fact that standard datasets such as KITTI and Waymo Open Dataset rely on fixed sensor configurations, limiting the analysis of observability from non-conventional viewpoints and in compact platforms.

This issue is especially relevant in compact autonomous platforms, where sensor placement is constrained by vehicle geometry, mounting availability, self-occlusion, field-of-view overlap, and cost [6,8,9]. A roof-mounted LiDAR usually provides wider contextual coverage and a more global perspective, but its predominantly top-down viewpoint may under-represent some pedestrian body features. In contrast, a lower-mounted side or corner sensor may better preserve frontal or lateral body structure, particularly in the torso and leg regions, although with more limited global coverage. These trade-offs suggest that LiDAR placement should be regarded as a measurable perception-design variable rather than as a secondary implementation detail [6,7,10].

A second gap concerns how pedestrian observability should be quantified. In many practical studies, sensing quality is implicitly judged through the number of returns, detector confidence, or downstream task performance [1,11,12]. Yet a larger number of points does not necessarily imply a more complete or more informative pedestrian representation. A point cloud may exhibit strong local density while still lacking sufficient projected support, volumetric occupancy, or balanced spatial coverage across the main body dimensions. This limitation is particularly important for pedestrians, whose LiDAR signatures are relatively sparse, deformable, and strongly viewpoint-dependent when compared with larger rigid objects [11,12,13]. Consequently, there is a need for an explicit object-level framework capable of distinguishing among local density, geometric extent, projected completeness, volumetric support, and structural balance [9,13].

Although LiDAR–camera fusion has become a prominent perception strategy in autonomous driving, a LiDAR-only analysis remains valuable when the objective is to isolate the geometric effects of sensor viewpoint and placement. Cameras provide semantic, texture, and appearance cues that are highly informative for classification and scene interpretation, whereas LiDAR provides direct three-dimensional geometric measurements and greater robustness to illumination changes. Recent advances in automatic LiDAR–camera calibration, including frameworks such as CalibRefine [14], further highlight the practical relevance of fusion-based perception while also showing that reliable fusion depends critically on accurate cross-sensor geometric alignment. From this perspective, LiDAR-only observability analysis should not be viewed as an alternative to multimodal perception, but rather as a complementary and more controlled approach for evaluating how much structurally reliable target geometry is captured before higher-level fusion and semantic interpretation are introduced.

### Main Contributions

The present study evaluates pedestrian observability using the ANTA autonomous vehicle prototype, a research platform developed at Universidad Politécnica Salesiana in Cuenca, Ecuador, for autonomous driving experimentation and multisensor perception studies [15,16,17]. Two sensing positions are analyzed: a roof-mounted Top LiDAR (TL) and a Front-Right LiDAR (FRL). Rather than directly benchmarking a learning-based detector, this work addresses a more transferable geometric question: *how much structural information each LiDAR contributes to the three-dimensional observability of a pedestrian at different distances, and how is that observability modified by multi-view fusion?* To answer this question, a controlled experimental framework is defined using point-cloud descriptors computed inside a canonical pedestrian-centered reference volume extracted from ROS 2 bag recordings.

The proposed framework combines geometric extent ratios, projected occupancy, voxel-based volumetric occupancy, and statistical descriptors of the local point distribution into a unified global observability score, $S_{3D}$. The underlying hypothesis is that pedestrian observability is strongly viewpoint-dependent and cannot be characterized by point density alone. More specifically, it is hypothesized that the TL and FRL sensors provide complementary geometric information, and that their fusion yields a more complete and balanced three-dimensional pedestrian representation than either individual viewpoint across the evaluated distance range, in line with current trends toward complementary multi-view perception [3,4,18].

The experiments were conducted with a human target positioned at longitudinal distances from 1 to 20 m relative to the vehicle, using synchronized point-cloud recordings from both sensing positions. The results show that very short distances maximize local sampling density, short-to-intermediate distances maximize projected and volumetric completeness, and intermediate distances provide the best overall balance between geometric extent and structural support. In particular, the fused configuration consistently produced the most robust observability profile, while the interval between 3 and 7 m emerged as the most favorable operating range for balanced pedestrian representation. These findings are relevant for LiDAR placement studies, multi-sensor configuration design, and perception-system development in autonomous driving applications, especially in compact platforms where sensor placement constraints directly affect pedestrian observability [6,8,9].

The main contributions of this paper are summarized as follows:

- A three-dimensional experimental framework for quantifying pedestrian observability on a multi-LiDAR autonomous vehicle platform using controlled distance-based measurements and a canonical pedestrian-centered reference volume.
- A unified set of geometric, projected, volumetric, and statistical descriptors for characterizing pedestrian representation, including extent ratios, projected surface occupancy, voxel-based volumetric occupancy, and a global three-dimensional observability score.
- A comparative evaluation of complementary LiDAR viewpoints, namely a roof-mounted Top LiDAR (TL), a Front-Right LiDAR (FRL), and their fused configuration, over longitudinal distances ranging from 1 to 20 m.
- Practical design evidence showing that complementary LiDAR viewpoints provide a more robust and physically meaningful pedestrian representation than single-view configurations, which is directly relevant for sensor-placement strategies in autonomous driving systems.

To position the proposed framework within the existing literature, Section 2 reviews recent advances in LiDAR-based pedestrian perception, with particular emphasis on sensor configuration, placement, and geometry-oriented observability limitations.

## 2. Related Work

Recent literature on LiDAR-based pedestrian perception can be organized into three complementary but distinct perspectives, as summarized in Table 1. The first is an *algorithm-driven* perspective, in which the main objective is to improve pedestrian detection, tracking, or scene understanding performance. The second is a *hardware- and placement-oriented* perspective, in which the emphasis is placed on sensor characteristics such as field of view, angular resolution, or mounting configuration. The third, which is the perspective adopted in the present work, is *geometry-driven observability*, where the central question is not primarily whether a detector performs well, but how completely and consistently the pedestrian geometry is represented as a function of LiDAR viewpoint and sensor configuration.

Most recent studies are dominated by the first of these perspectives. A large body of work focuses on improving downstream perception performance through deep learning architectures, including CNN-based and point-based models such as VoxelNet, PointNet, and PV-RCNN, as well as more advanced strategies designed to address occlusion and corner cases through meta-learning or hybrid detection frameworks [23,24,25,30,31]. In parallel, several multimodal approaches integrate LiDAR with cameras, radar, and infrared sensors to improve robustness under challenging environmental conditions and partial observability scenarios [19,20,21,22,32,33,34,35,36,37]. These studies have demonstrated important gains in detection accuracy and reliability, especially in complex urban environments. However, their primary emphasis remains on detector- or task-level performance, and therefore the geometric completeness of the sensed pedestrian representation is usually treated only implicitly rather than as an explicit object of analysis.

A related group of studies has explored geometric aspects through reconstruction and point-cloud completion methods, often using deep learning to recover missing structural information [26,27,28,29]. These works are relevant because they introduce geometric evaluation criteria such as Chamfer Distance, IoU, and RMSE. Nevertheless, their objective is fundamentally different from that of the present study. Rather than analyzing how sensor viewpoint affects what is directly observed, they seek to infer or reconstruct information that is not fully captured in the original data. As a result, they contribute to geometry recovery, but they do not explicitly quantify pedestrian observability as a function of LiDAR placement.

The second major perspective is hardware- and placement-oriented. In this line, several studies have examined LiDAR sensing from the standpoint of field of view (FOV), angular resolution, scanning mechanisms, coverage, and sensor arrangement [38,39,40,41,42]. These works provide valuable design insights and show that sensor geometry strongly influences perception capability. However, they typically evaluate sensor characteristics at the hardware level, often through coverage- or projection-based criteria, without establishing an explicit object-level framework linking sensor placement to the structural completeness of pedestrian representation. In other words, they help explain how sensors differ, but only in a limited way address how those differences translate into the three-dimensional observability of a pedestrian target.

From an evaluation perspective, most existing studies still rely on task-driven metrics such as mean Average Precision (mAP), Intersection over Union (IoU), Average Displacement Error (ADE), and Final Displacement Error (FDE) [43,44,45,46,47,48,49,50,51]. These metrics are effective for benchmarking detection, prediction, and tracking pipelines, but they do not directly capture structural properties such as volumetric completeness, projected support, internal spatial balance, or multi-view geometric complementarity. Consequently, although the literature offers extensive evidence on algorithmic performance and sensor behavior, it still leaves open a prior geometry-centered question: how LiDAR viewpoint configuration shapes the structural representation of pedestrians before downstream perception modules are applied.

In this context, the present work adopts a geometry-driven observability perspective. Using a controlled real-world benchmark, it evaluates how a roof-mounted Top LiDAR (TL), a Front-Right LiDAR (FRL), and their fused configuration affect the structural representation of pedestrians across distance. Rather than optimizing a detector or comparing learning architectures, the proposed framework quantifies observability through projection-based, volumetric, and statistical descriptors, together with robustness- and complementarity-oriented indices. This perspective differentiates the present study from the existing literature by shifting the focus from task-level performance or hardware specifications to the explicit analysis of how LiDAR viewpoint configuration determines the geometric completeness and balance of pedestrian point clouds. Based on this identified gap, Section 3 describes the experimental platform, the pedestrian-centered reference formulation, and the observability metrics used to evaluate the TL, FRL, and fused configurations.

## 3. Materials and Methods

### 3.1. Experimental Platform

**Figure 1:**
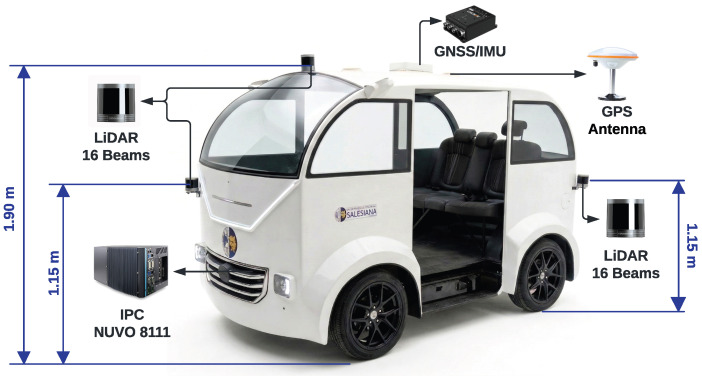
ANTA autonomous vehicle prototype and multisensor configuration used for the LiDAR observability analysis.

The experimental campaign was conducted using the ANTA autonomous vehicle prototype, a laboratory-scale research platform equipped with multiple perception and positioning sensors, including LiDAR units, GNSS/GPS receivers, antennas, and an onboard Nuvo-8111 industrial computer, as shown in Figure 1. The platform operates within a ROS 2-based sensing and recording architecture that supports environmental perception, localization, mapping, and autonomous driving validation under both controlled and real-world operating conditions.

Although the vehicle is equipped with three RSHELIOS 16P LiDAR sensors installed at different mounting positions, the present observability benchmark focuses on two of them: a roof-mounted Top LiDAR (TL), located 1.90 m above ground level, and a Front-Right LiDAR (FRL), mounted at 1.15 m. These sensing positions were selected because they provide complementary views of the pedestrian target. Specifically, the TL offers a higher and more global perspective, whereas the FRL provides a lower and more frontal–lateral viewpoint. Their combined use therefore enables the evaluation of multi-view fusion for pedestrian observability.

### 3.2. ROS 2 Bag Acquisition and Data Processing

During the experiments, multisensor data were recorded directly on the onboard computer in ROS 2 bag format. These recordings constituted the raw experimental source used both for map generation and for the subsequent LiDAR-based observability analysis, following an offline processing strategy similar to that adopted in related studies [52,53].

For the pedestrian-observability benchmark, the recorded datasets were transferred to MATLAB for offline processing. The ROS 2 bags were loaded using ros2bagreader, and the point clouds associated with the topics /rs/points and /rs/points_right were extracted for the TL and FRL sensors, respectively. For each evaluated distance, a representative frame index *k* was selected from the recorded sequence, and the corresponding point-cloud message was decoded into Cartesian coordinates using rosReadXYZ. Samples containing non-finite values were removed prior to further processing.

This procedure enabled a reproducible offline evaluation of point-cloud coverage, observability metrics, and statistical descriptors from the recorded ROS 2 datasets. Moreover, the TL and FRL point clouds were processed independently so that the viewpoint-specific characteristics of each sensor were preserved prior to the fusion stage.

In addition to the pedestrian-observability benchmark, the multisensor recordings were also used to support the georeferenced deployment of the ANTA platform in the experimental environment. A LIO-SAM-based workflow combined LiDAR, IMU, and RTK-corrected GNSS/GPS measurements to generate a consistent point-cloud map and a Lanelet-based road map for Autoware Universe integration. Since this mapping stage only provides deployment context and is not part of the observability metric formulation, the detailed workflow is provided in Appendix A.1.

### 3.3. Pedestrian ROI Extraction and Local Reference Frame

For each distance condition, a three-dimensional region of interest (ROI) was manually defined to isolate the pedestrian target and remove irrelevant surrounding points. The extraction ROI was expressed as

(1) $${\mathrm{ROI}}_{obj}=\left[x_{\min},x_{\max},y_{\min},y_{\max},z_{\min},z_{\max}\right],$$

where *x* denotes the longitudinal axis relative to the vehicle, *y* the lateral axis, and *z* the vertical axis. The same ROI extraction procedure was independently applied to the TL and FRL point clouds, and the resulting target subsets were subsequently merged to construct the fused configuration.

**Figure 2:**
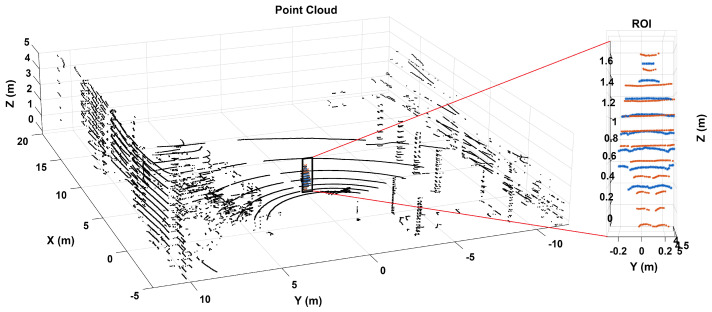
ROI selection procedure used in the pedestrian observability analysis. The left panel shows the original point cloud, while the right panel presents a zoomed view of the selected region of interest containing the pedestrian target.

Figure 2 illustrates the ROI selection procedure used in this study. The left panel shows the original point cloud acquired during the experiment, while the right panel presents a magnified view of the selected ROI containing the pedestrian target. This step ensured that the subsequent observability analysis was restricted to points associated with the human subject.

The pedestrian target was evaluated at multiple longitudinal distances with respect to the vehicle, namely 1, 2, 3, 4, 5, 6, 7, 8, 9, 10, 15, and 20 m. For each distance, representative ROS 2 frames were selected from the recorded sequences to compare the target representation obtained from TL, FRL, and the fused configuration.

To reduce the influence of small placement variations during the experiments, the geometric analysis was not performed directly in the vehicle frame. Instead, after ROI extraction, the point clouds were expressed in a common pedestrian-centered local reference frame. The horizontal coordinates were recentered using a common target reference $\left(x_{c},y_{c}\right)$, whereas the vertical coordinate was referenced to the pedestrian base level $z_{b}$. Thus, the local coordinates were defined as

(2) $$x_{i}^{\ell }=x_{i}-x_{c},\;y_{i}^{\ell }=y_{i}-y_{c},\;z_{i}^{\ell }=z_{i}-z_{b}.$$

This transformation enabled all sensing configurations to be evaluated within the same canonical pedestrian volume, thereby reducing the sensitivity of the analysis to slight target-positioning deviations across distance conditions.

**Figure 3:**
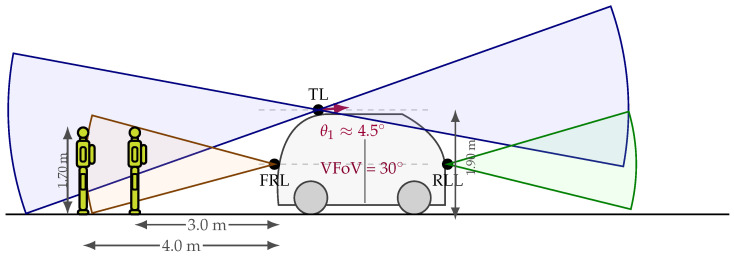
Side-view schematic of the LiDAR arrangement on the ANTA prototype. The roof-mounted Top LiDAR (TL), Front-Right LiDAR (FRL), and Rear-Left LiDAR (RLL) are shown together with their installation heights, vertical fields of view, and the nominal pedestrian dimensions. Although the platform includes three LiDAR units, the quantitative observability analysis presented in this study focuses on TL, FRL, and their fused configuration.

The nominal pedestrian dimensions used in the study were $L_{x}=0.30\;m$, $L_{y}=0.60\;m$, and $L_{z}=1.70\;m$, corresponding to body thickness, width, and height, respectively. Figure 3 provides the geometric context of the analysis by showing the relative arrangement of the TL and FRL sensors with respect to the vehicle and the pedestrian target.

**Figure 4:**
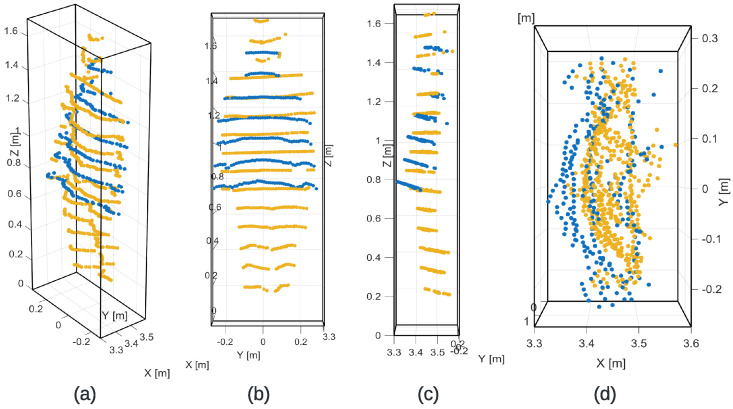
Distribution of the pedestrian point cloud inside the defined ROI from multiple viewpoints: (**a**) 3D perspective view, (**b**) frontal projection on the *Y*–*Z* plane, (**c**) lateral projection on the *X*–*Z* plane, and (**d**) top projection on the *X*–*Y* plane.

Let $P={\left\{\left(x_{i},y_{i},z_{i}\right)\right\}}_{i=1}^{N}$ denote the set of LiDAR points associated with the pedestrian target after ROI extraction [54,55,56]. In contrast to the previous 2D formulation, the pedestrian was modeled here as a three-dimensional body approximation with nominal dimensions $L_{x}=0.30\;m$, $L_{y}=0.60\;m$, and $L_{z}=1.70\;m$, corresponding to thickness, width, and height, respectively. Figure 4 illustrates the spatial distribution of the point cloud within the pedestrian ROI from multiple viewpoints. This formulation allows the observability analysis to explicitly account for the spatial extent of the target in all three dimensions [57].

### 3.4. Three-Dimensional Coverage and Observability Metrics

The local target volume was defined as

(3) $$V_{ped}=\left[-\frac{L_{x}}{2},\frac{L_{x}}{2}\right]\times \left[-\frac{L_{y}}{2},\frac{L_{y}}{2}\right]\times \left[0,L_{z}\right].$$

Only the points lying inside $V_{ped}$ were used to compute the three-dimensional observability metrics.

#### 3.4.1. 3D Extent Ratios

To quantify the geometric extent of the observed target, three normalized span-based indicators were defined along the three principal axes:

(4) $$C_{x}=\min\left(\frac{\max\left(x_{i}^{\ell }\right)-\min\left(x_{i}^{\ell }\right)}{L_{x}},1\right),$$

(5) $$C_{y}=\min\left(\frac{\max\left(y_{i}^{\ell }\right)-\min\left(y_{i}^{\ell }\right)}{L_{y}},1\right),$$

(6) $$C_{z}=\min\left(\frac{\max\left(z_{i}^{\ell }\right)-\min\left(z_{i}^{\ell }\right)}{L_{z}},1\right).$$

These three ratios quantify the fraction of the nominal pedestrian thickness, width, and height effectively represented by the LiDAR returns.

#### 3.4.2. Projected Surface Coverage

Although the pedestrian was modeled as a three-dimensional target, projected surface coverage remained an informative descriptor because it reveals how LiDAR returns are distributed over the different visible faces of the body. Specifically, complementary orthogonal projections allow the analysis to capture not only the frontal–lateral body shape, but also the depth-related and footprint-related spatial support of the target [2,58]. For this reason, the local point cloud was projected onto the three principal planes: *Y*–*Z*, *X*–*Z*, and *X*–*Y*. Each projection was discretized using a grid step of 0.05 m, corresponding to a cell area of $0.0025\;m^{2}$.

Let $N_{occ}^{yz}$, $N_{occ}^{xz}$, and $N_{occ}^{xy}$ denote the number of occupied cells in the corresponding projected domains, and let $N_{tot}^{yz}$, $N_{tot}^{xz}$, and $N_{tot}^{xy}$ denote the total number of cells in each projection. The projected occupancy ratios were defined as

(7) $$C_{yz}=\frac{N_{occ}^{yz}}{N_{tot}^{yz}},$$

(8) $$C_{xz}=\frac{N_{occ}^{xz}}{N_{tot}^{xz}},$$

(9) $$C_{xy}=\frac{N_{occ}^{xy}}{N_{tot}^{xy}}.$$

Here, $C_{yz}$ characterizes the lateral–vertical body surface, $C_{xz}$ describes the depth–height coverage, and $C_{xy}$ represents the horizontal footprint of the pedestrian target. Together, these ratios provide complementary information on how completely the body is represented from different geometric viewpoints.

To obtain a single descriptor of projected surface completeness, a weighted combined surface-coverage index was defined as

(10) $$C_{surf}=w_{yz}C_{yz}+w_{xz}C_{xz}+w_{xy}C_{xy},$$

with

(11) $$w_{yz}+w_{xz}+w_{xy}=1.$$

In this study, the selected values were $w_{yz}=0.50$, $w_{xz}=0.25$, and $w_{xy}=0.25$. A larger weight was assigned to the *Y*–*Z* projection because it provides the most informative frontal–lateral representation of the pedestrian, preserving the two most distinctive body dimensions for LiDAR-based perception, namely height and lateral extent. By contrast, the *X*–*Z* and *X*–*Y* projections were assigned lower but equal weights, since they contribute complementary information on body thickness and horizontal support while being generally less discriminative than the *Y*–*Z* view for pedestrian observability assessment.

Accordingly, $C_{surf}$ was defined as a task-oriented projected completeness descriptor in which the frontal–lateral body representation plays the dominant role, while the remaining two projections preserve the three-dimensional consistency of the evaluation. A sensitivity analysis was also considered to verify that moderate variations in these projection weights did not modify the main comparative trends among TL, FRL, and the fused configuration.

#### 3.4.3. Volumetric Occupancy

To quantify how completely the pedestrian body was sampled in 3D, the local target volume $V_{ped}$ was discretized into cubic voxels of side length 0.05 m. Let $N_{vox}^{occ}$ be the number of occupied voxels and $N_{vox}^{tot}$ the total number of voxels inside the canonical pedestrian volume [2,13,59,60]. The volumetric occupancy ratio was then defined as

(12) $$C_{vol}=\frac{N_{vox}^{occ}}{N_{vox}^{tot}}.$$

This metric provides a direct estimate of how much of the nominal 3D pedestrian body is effectively intersected by LiDAR returns.

In addition to the coverage descriptors, the total number of target points $N_{pts}$, the number of occupied cells or voxels, and the number of contributing scan layers were retained as complementary indicators of target observability. The number of scan layers was estimated from the distinct vertical angle bins associated with the target returns. Together, these quantities reflect the sampling density and structural support available for pedestrian perception.

#### 3.4.4. Global 3D Observability Score

To summarize the previous descriptors into a single indicator, a global three-dimensional observability score was defined as a weighted linear combination of the normalized extent, projected occupancy, and volumetric occupancy terms:

(13) $$S_{3D}=w_{x}C_{x}+w_{y}C_{y}+w_{z}C_{z}+w_{s}C_{surf}+w_{v}C_{vol},$$

where $w_{x}+w_{y}+w_{z}+w_{s}+w_{v}=1$. This formulation follows the weighted-sum scalarization principle commonly used in multi-criteria optimization to aggregate several complementary indicators into a single scalar objective. In the present case, the score integrates geometric extent along the three spatial directions, projected surface completeness, and volumetric occupancy, thereby providing a compact descriptor of pedestrian observability under each sensing configuration. The use of such a composite score is further motivated by prior studies showing that LiDAR placement affects perception quality from multiple geometric perspectives, as well as by occupancy-based representations that model spatial completeness in voxelized 3D space [13,61,62].

It should be noted that $S_{3D}$ is introduced in this work as a task-oriented composite descriptor rather than as a universal metric. Its purpose is to summarize complementary observability properties into a single interpretable score while preserving the individual metrics for detailed analysis.

The weighting coefficients were selected according to a task-oriented expert criterion within a weighted-sum multi-criteria formulation. Since all component metrics were normalized to the interval [0,1], the weights directly represent their relative importance in the final observability score. A larger weight was assigned to $C_{z}$ because pedestrian height provides one of the most distinctive and stable geometric cues in LiDAR-based perception. Intermediate weights were assigned to $C_{y}$, $C_{surf}$, and $C_{vol}$, as these descriptors capture lateral extent and spatial completeness of the observed body. A slightly lower weight was assigned to $C_{x}$, because target thickness is more difficult to observe consistently from limited LiDAR viewpoints and is more sensitive to minor target-placement variations. Accordingly, the selected values were $w_{x}=0.15$, $w_{y}=0.20$, $w_{z}=0.25$, $w_{s}=0.20$, and $w_{v}=0.20$. In addition, a sensitivity analysis was considered to verify that moderate variations in the weighting coefficients did not alter the main ranking trends among TL, FRL, and the fused configuration.

#### 3.4.5. Statistical Characterization of the Local Point Distribution

To complement the coverage analysis, descriptive statistics of the point distribution were computed within the canonical pedestrian volume. These included the centroid coordinates $\left(\mu_{x},\mu_{y},\mu_{z}\right)$, the variances $\left(\sigma_{x}^{2},\sigma_{y}^{2},\sigma_{z}^{2}\right)$, and the standard deviations $\left(\sigma_{x},\sigma_{y},\sigma_{z}\right)$ in the local pedestrian frame. In addition, occupancy ratios were computed for the three projected planes and for the voxelized 3D volume [63,64].

To characterize density uniformity, voxel-count descriptors were also calculated, including the mean, variance, and standard deviation considering all voxels, as well as the corresponding statistics restricted to occupied voxels only. These descriptors were used to assess not only how much of the pedestrian target was covered, but also how LiDAR returns were spatially distributed inside the nominal body volume.

### 3.5. Fusion Strategy

To evaluate the potential benefit of multi-view sensing, a fused configuration was constructed by merging the pedestrian point subsets extracted from TL and FRL for the same distance condition. Fusion was performed after the initial target extraction step so that only pedestrian-related points from each sensor were retained. A common pedestrian-centered local reference frame was then defined and applied consistently to the TL, FRL, and fused point clouds. This ensured that all observability metrics and statistical descriptors were computed inside the same canonical pedestrian volume, thereby enabling a fair comparison across sensing configurations.

The same set of three-dimensional extent ratios, projected surface occupancy indices, volumetric occupancy measures, density descriptors, and statistical indicators was subsequently computed for the fused point cloud. In this way, the proposed fusion strategy quantifies the gain provided by complementary LiDAR viewpoints not only in terms of point count, but also in terms of geometric completeness and volumetric target representation.

## 4. Results

Following the experimental protocol and the three-dimensional observability framework described in Section 3, the pedestrian target was evaluated at longitudinal distances of 1, 2, 3, 4, 5, 6, 7, 8, 9, 10, 15, and 20 m using three sensing configurations: the Top LiDAR (TL), the Front-Right LiDAR (FRL), and their fused point cloud.

The complete numerical results are reported in Appendix A.2 in four complementary tables (Table A1, Table A2, Table A3, Table A4 and Table A5). Table A1 summarizes the main three-dimensional geometric extent and coverage descriptors; Table A2 reports the associated occupancy indicators and the global observability score; and Table A3 and Table A4 present the statistical characterization of the local point distribution and the voxel-based descriptors, respectively.

Together, these results provide a comprehensive basis for evaluating not only the geometric completeness of the pedestrian representation, but also the internal spatial distribution and density of LiDAR returns within the canonical target volume defined in Section 3. The analysis is organized below to progressively examine distance-dependent observability, sensor complementarity, projected and volumetric coverage, and the statistical structure of the local point distribution.

### 4.1. Distance-Dependent 3D Observability

The evolution of the proposed observability metrics as a function of distance reveals clear differences between sensing configurations and highlights the benefits of multi-view perception.

In general, the fused configuration provides the most balanced three-dimensional representation of the pedestrian, while the individual LiDAR sensors exhibit complementary strengths. When averaged over all evaluated distances, the fused configuration achieves the highest global observability score, with a mean value of 0.563, compared with 0.504 for FRL and 0.432 for TL (Table A2). This result confirms that combining top and frontal–lateral viewpoints improves the overall completeness of the pedestrian representation within the local target frame.

**Figure 5:**
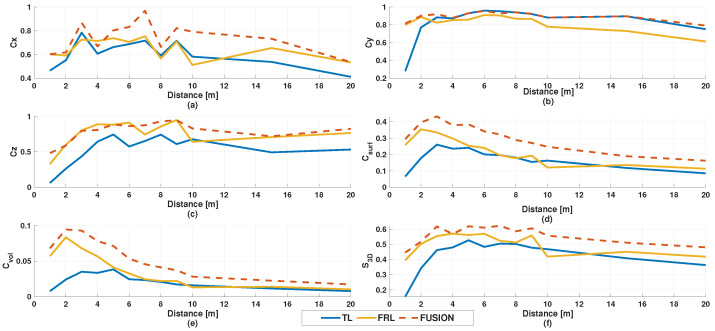
Distance-dependent behavior of the main observability metrics for TL, FRL, and the fused configuration: (**a**) $C_{x}$, (**b**) $C_{y}$, (**c**) $C_{z}$, (**d**) $C_{surf}$, (**e**) $C_{vol}$, and (**f**) $S_{3D}$.

Figure 5 illustrates the distance-dependent behavior of the main observability descriptors. The fused configuration consistently maintains higher and more stable values across most metrics, particularly for projected surface coverage, volumetric occupancy, and the global score.

A key observation is that the optimal distance depends on the specific descriptor being considered. The highest global observability score was obtained for the fused configuration at 7 m, with $S_{3D}=0.624$. This maximum corresponds to a well-balanced geometric representation, characterized by high values of $C_{x}=0.969$, $C_{y}=0.928$, and $C_{z}=0.875$, indicating that the overall spatial extent of the pedestrian body is most completely captured at this distance.

In contrast, the maximum volumetric occupancy was achieved at a shorter distance, namely 2 m for the fused configuration, with $C_{vol}=0.0948$, while the highest projected surface coverage occurred at 3 m, with $C_{surf}=0.433$. These results indicate that different aspects of observability—such as geometric extent, surface completeness, and volumetric filling—are maximized at different distance regimes. Therefore, no single distance simultaneously optimizes all descriptors; instead, the results reveal a transition from dense local sampling at short ranges to more balanced geometric observability at intermediate ranges.

**Figure 6:**
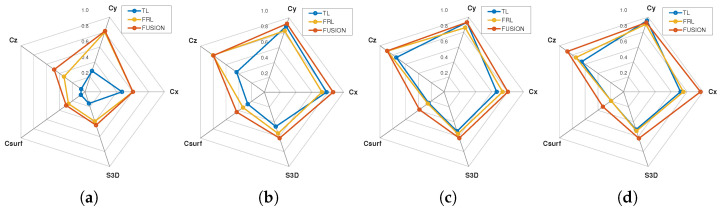
Radar-chart comparison of the main observability descriptors for TL, FRL, and the fused configuration at representative distances: (**a**) 1 m, (**b**) 3 m, (**c**) 5 m, and (**d**) 7 m. The plots provide a compact multimetric summary of geometric extent, projected completeness, and global observability, highlighting the transition from dense short-range sampling to the more balanced fused representation achieved at intermediate distances.

To further synthesize the multimetric behavior of the three sensing configurations, radar-chart representations were generated at four representative distances (1, 3, 5, and 7 m), as shown in Figure 6. These plots compactly summarize the relative balance among the main observability descriptors and make the multimetric contribution of each sensing configuration visually explicit.

At 1 m, the radar profiles remain uneven rather than uniformly expanded, reflecting high local sampling density but limited overall completeness, particularly in the volumetric-related descriptors. At 3 m, the profiles expand noticeably, especially for the fused configuration, indicating improved projected coverage and a more complete intersection with the pedestrian body. At 5 m, the radar shapes remain stable, suggesting that the representation still preserves a broad geometric extent despite the gradual reduction in point density. At 7 m, the fused configuration exhibits the most balanced overall profile, with simultaneously high values of geometric extent, surface coverage, and global observability. This visual summary is fully consistent with the numerical results and reinforces the interpretation that the main benefit of fusion lies not in a single descriptor, but in the overall balance achieved across multiple complementary observability metrics.

### 4.2. Comparison of Individual and Fused Sensing Configurations

The comparative analysis of TL, FRL, and the fused configuration highlights the complementary nature of the two LiDAR viewpoints and the consistent advantage of multi-view sensing within the three-dimensional observability framework defined in Section 3.

**Figure 7:**
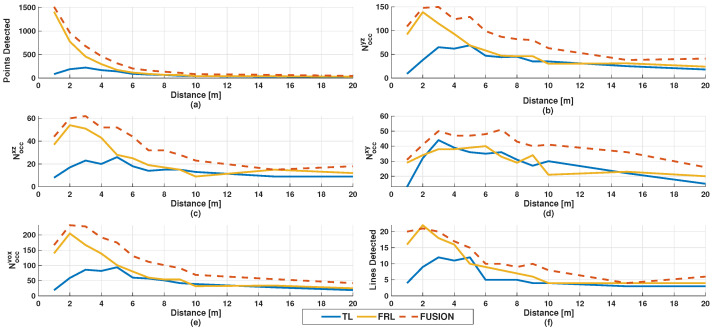
Distance-dependent evolution of occupancy-related descriptors for TL, FRL, and the fused configuration: (**a**) number of detected points, (**b**) $N_{occ}^{yz}$, (**c**) $N_{occ}^{xz}$, (**d**) $N_{occ}^{xy}$, (**e**) $N_{occ}^{vox}$, and (**f**) number of detected scan layers.

Figure 7 presents the evolution of occupancy-related descriptors as a function of distance, including point counts, projected occupancy, voxel occupancy, and scan-layer support. These indicators provide additional insight into how the spatial support of the pedestrian representation varies across sensing configurations.

The fused point cloud consistently produces the highest values for most occupancy-based metrics, including the maxima of $C_{yz}$, $C_{xz}$, $C_{xy}$, $C_{surf}$, $C_{vol}$, and the corresponding occupied-cell counts. In particular, the fused configuration achieves its highest projected occupancy at 3 m, with $C_{yz}=0.368$ and $C_{xz}=0.304$, and its highest horizontal footprint coverage at 7 m, with $C_{xy}=0.708$. Similarly, the maximum number of occupied voxels is obtained at 2 m, with $N_{occ}^{vox}=232$.

Despite this overall dominance, the individual sensors exhibit distinct strengths. The FRL sensor is particularly effective in capturing vertical structure, achieving the highest single-sensor value of $C_{z}$ at 9 m ($C_{z}=0.948$), as well as the largest number of contributing scan layers at 2 m (22 layers). This behavior is consistent with its frontal–lateral viewpoint, which provides improved intersection with the vertical body profile.

In contrast, the TL sensor shows its strongest performance in lateral coverage, reaching the highest single-sensor value of $C_{y}$ at 6 m ($C_{y}=0.960$). The elevated viewpoint therefore remains advantageous for preserving body width and complementing the frontal sensor in the fused configuration.

From a practical perspective, the fusion gain is positive at 11 of the 12 evaluated distances. On average, the fused configuration improves the global observability score by 0.055 absolute units, corresponding to an average relative gain of 11.1% over the best single-sensor alternative. The most pronounced improvement is observed at 7 m, where fusion outperforms the best individual sensor by 0.099 (18.95%), while the only marginal exception occurs at 4 m, where FRL slightly exceeds the fused configuration by 0.0015. These results confirm that the proposed fusion strategy provides a robust and consistent improvement across a wide range of operating distances.

Table 2 consolidates the previous observations by identifying the best-performing configuration for each evaluated metric. A clear dominance of the fused configuration can be observed, as it achieves the highest values in most geometric and occupancy-related descriptors, including $C_{x}$, $C_{yz}$, $C_{xz}$, $C_{xy}$, $C_{surf}$, $C_{vol}$, and the global score $S_{3D}$.

In particular, the fused configuration reaches the maximum global observability score at 7 m, confirming that this distance provides the most balanced combination of geometric extent and spatial completeness. Similarly, the maximum volumetric occupancy and voxel-based descriptors are obtained at short distances (2–3 m), indicating that dense sampling conditions favor volumetric reconstruction of the pedestrian body.

Nevertheless, the individual sensors achieve the best performance in specific descriptors. The TL sensor provides the highest lateral coverage ($C_{y}$), while the FRL sensor achieves the highest vertical extent ($C_{z}$) and scan-layer support. These results reinforce the interpretation that the two LiDAR viewpoints capture complementary aspects of the pedestrian geometry.

Overall, Table 2 confirms that multi-view fusion not only increases the number of observed points, but also improves the geometric completeness and spatial distribution of the pedestrian representation. This behavior is fully consistent with the trends observed in the distance-dependent analysis and further supports the effectiveness of the proposed fusion strategy.

### 4.3. Projected and Volumetric Coverage Trends

The projected coverage descriptors provide additional insight into how the visible pedestrian surface evolves with distance. In particular, the *Y*–*Z* projection, which represents the most informative frontal–lateral view of the body, showed the clearest benefit from fusion. The occupancy ratio in this plane increased from 0.277 at 1 m to a maximum of 0.392 at 3 m for the fused configuration, and then progressively decreased as distance increased. A similar trend was observed for $C_{xz}$ and $C_{vol}$, which peaked at short and intermediate distances and then decayed at larger ranges. These trends indicate that the densest and most spatially complete representations are achieved when the pedestrian is sufficiently close to generate dense returns, but not so close that the body is only partially intercepted by the available beams.

**Figure 8:**
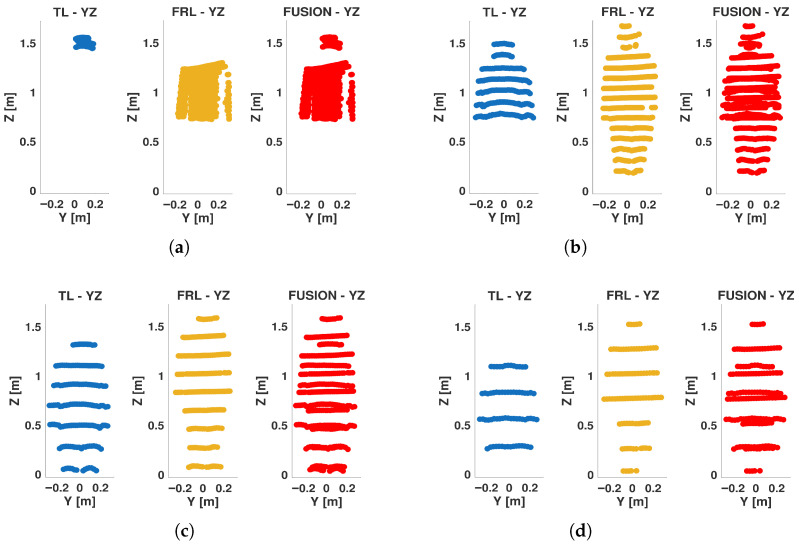
Representative frontal-plane (*Y*–*Z*) point-cloud distributions of the pedestrian target at different distances: (**a**) 1 m, (**b**) 3 m, (**c**) 5 m, and (**d**) 7 m. These examples illustrate how the complementary contributions of TL and FRL combine to form the fused representation and how projected coverage evolves from dense short-range sampling to more balanced intermediate-range observability.

The visual examples in Figure 8 support the numerical trends observed in the projected metrics. At 1 m, the point cloud is dense but spatially concentrated, indicating strong local sampling with limited global body coverage. At 3 m, the fused representation exhibits a clearer and more complete frontal silhouette, which is consistent with the maximum values of projected occupancy and combined surface coverage. At 5 m, the visible outline remains well preserved, although the sampling density begins to decrease. At 7 m, the projected representation still retains a coherent body shape while providing a more balanced distribution between density and extent, consistent with the maximum global observability score.

The combined surface descriptor $C_{surf}$ showed a related but not identical behavior. While it was highest at 3 m for FUSION, it remained comparatively stable across the 3–10 m range, demonstrating that the target retained a relatively consistent projected representation even when volumetric filling declined. This finding is especially relevant for perception tasks that rely more on the visible outline and body envelope than on full volumetric completeness.

### 4.4. Statistical Characterization of the Local Point Distribution

The statistical descriptors computed in the local pedestrian-centered frame provide complementary insight into the internal structure of the LiDAR point distribution, extending the geometric and occupancy-based analysis presented in the previous subsections. In particular, the Gaussian approximations derived from the mean and standard deviation values allow the spatial distribution of LiDAR returns to be interpreted along each principal axis.

Across all evaluated distances, the horizontal centroids ($\mu_{x}$, $\mu_{y}$) remained close to zero for all sensing configurations, confirming that the adopted canonical reference frame effectively normalizes the pedestrian position. This spatial consistency ensures that the observed variations in the probability density functions (PDFs) are primarily driven by sensing geometry and not by misalignment effects. These Gaussian profiles should be interpreted as parametric approximations of the local point distribution derived from the reported mean and standard deviation values, rather than as empirical probability densities of the raw point-cloud samples.

**Figure 9:**
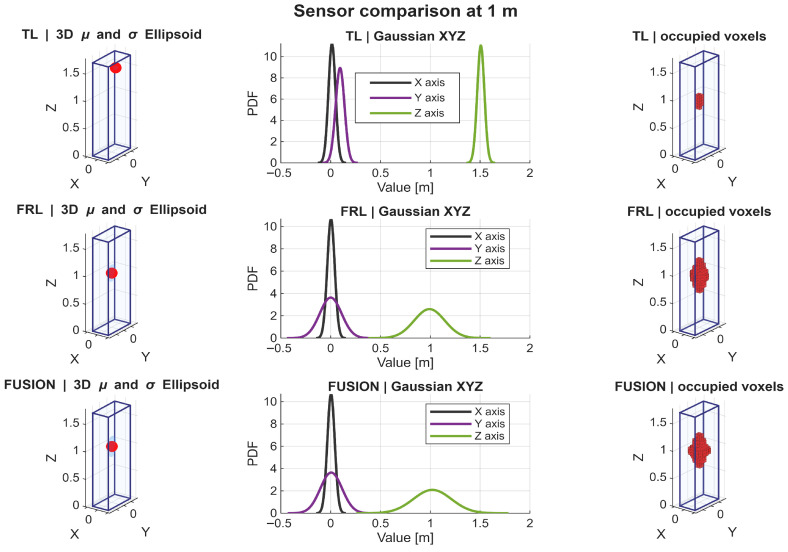
Statistical representation at 1 m for TL, FRL, and the fused configuration. The figure shows the centroid location in the canonical pedestrian volume (**left**), the Gaussian PDF comparison along the *X*, *Y*, and *Z* axes (**center**), and the schematic voxel occupancy representation (**right**).

A detailed analysis of the Gaussian distributions at representative distances of 1, 3, 5, and 7 m reveals a clear evolution of the spatial distribution of points. At 1 m, the PDFs along all axes exhibit narrow and sharply peaked profiles, indicating very high point concentration, as shown in Figure 9. In particular, the distribution along the *Z* axis shows a relatively limited spread, reflecting partial vertical coverage due to the proximity of the target, where only a subset of the body is effectively intercepted by the LiDAR beams. Similarly, the distributions along *X* and *Y* are tightly concentrated, indicating that most returns are clustered in a reduced spatial region. Therefore, although the nearest distance provides the highest local density, it does not yet produce the most spatially complete representation.

**Figure 10:**
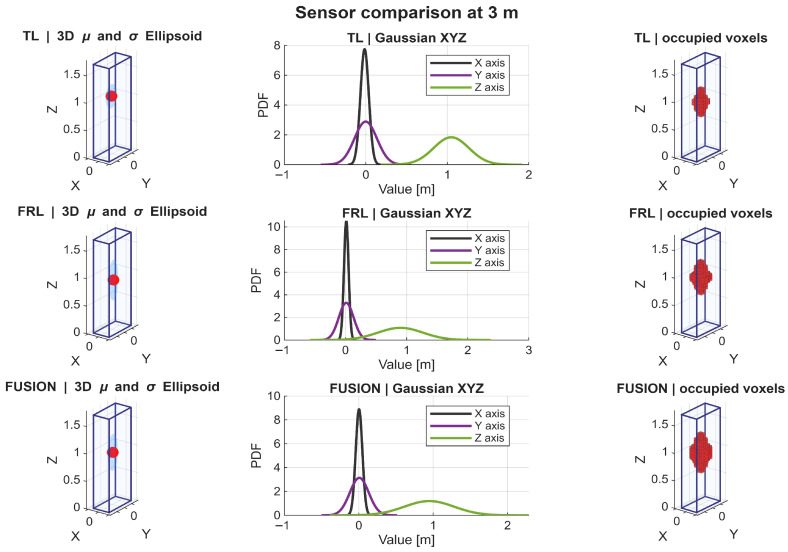
Statistical representation at 3 m for TL, FRL, and the fused configuration. The figure shows the centroid location in the canonical pedestrian volume (**left**), the Gaussian PDF comparison along the *X*, *Y*, and *Z* axes (**center**), and the schematic voxel occupancy representation (**right**).

At 3 m, the Gaussian profiles become wider, especially along the *Y* and *Z* axes. This increase in dispersion reflects a more complete intersection between the LiDAR beams and the pedestrian body, resulting in improved lateral coverage and a more representative vertical span. The PDFs at this distance show a balanced shape, with moderate peak values and increased spread, which is consistent with the observed maxima in projected coverage ($C_{surf}$) and occupancy-ratio-based statistical descriptors, as shown in Figure 10. In fact, the fused configuration reached the highest values of OccRatioYZ=0.3922, OccRatioXZ=0.3431, and OccRatioVox=0.1005 at 3 m, confirming that this distance provides the most spatially distributed filling of the canonical target volume.

**Figure 11:**
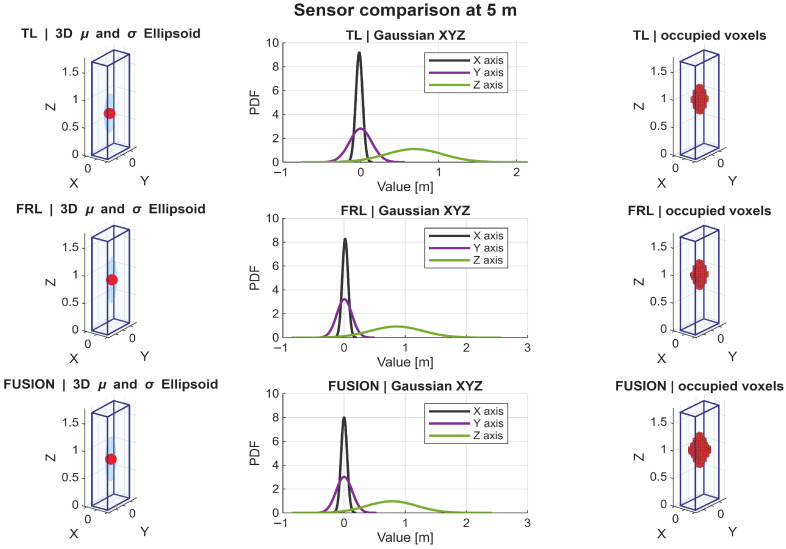
Statistical representation at 5 m for TL, FRL, and the fused configuration. The figure shows the centroid location in the canonical pedestrian volume (**left**), the Gaussian PDF comparison along the *X*, *Y*, and *Z* axes (**center**), and the schematic voxel occupancy representation (**right**).

At 5 m, the distributions maintain a relatively stable spread, but the peak values of the PDFs begin to decrease, indicating a reduction in local point density. However, the spatial extent along all axes remains well preserved. The *Z*-axis distribution, in particular, continues to exhibit a broad profile, suggesting that the vertical structure of the pedestrian is still adequately captured despite the reduction in point density, as shown in Figure 11.

**Figure 12:**
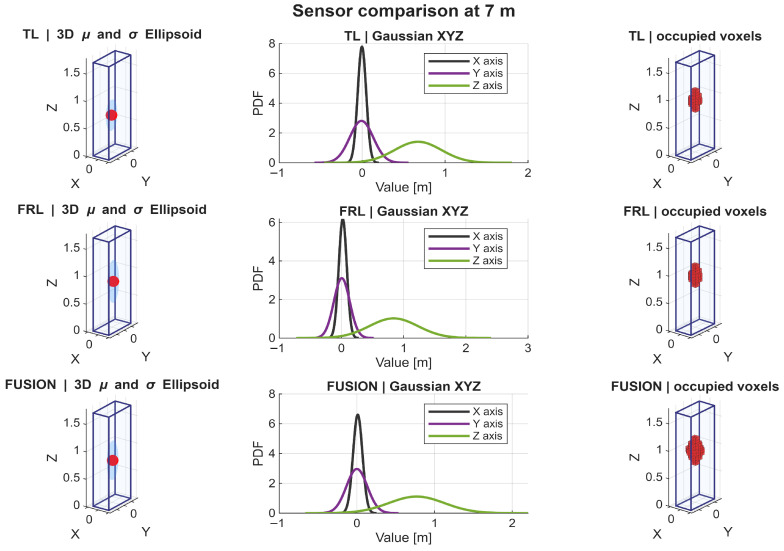
Statistical representation at 7 m for TL, FRL, and the fused configuration. The figure shows the centroid location in the canonical pedestrian volume (**left**), the Gaussian PDF comparison along the *X*, *Y*, and *Z* axes (**center**), and the schematic voxel occupancy representation (**right**).

At 7 m, the Gaussian distributions show the most balanced configuration. Although the peaks of the PDFs are lower—reflecting reduced point density—the spread along *X*, *Y*, and *Z* remains sufficiently large to preserve the geometric proportions of the pedestrian. This balance between dispersion and density is consistent with the maximum global observability score ($S_{3D}$) observed at this distance, as shown in Figure 12. In addition, the fused configuration achieved the highest horizontal-footprint occupancy ratio at this distance, with OccRatioXY=0.7083, which further supports the interpretation of 7 m as the most balanced operating point.

The statistical descriptors also highlight the complementary behavior of the two individual sensors. At very short distance, TL produced the highest vertical centroid, with MeanZ=1.5074 at 1 m, indicating a stronger concentration of returns on the upper body. By contrast, FRL yielded the highest vertical dispersion, with StdZ=0.5086 at 9 m, confirming its stronger ability to preserve the vertical body span. Likewise, FRL produced the highest statistics restricted to occupied voxels, with maximum values of MeanVoxelOcc=9.6104, VarVoxelOcc=52.3157, and StdVoxelOcc=7.2330 at 1 m, indicating highly concentrated local sampling in the occupied region. In contrast, the fused configuration produced the highest global voxel-density-related statistics, with MeanVoxelAll=0.6405, VarVoxelAll=8.9386, and StdVoxelAll=2.9898 at 1 m, reflecting the densest overall sampling of the canonical volume.

Taken together, these statistical results reinforce the conclusions derived from the geometric and occupancy-based metrics. Very short distances maximize local density, short-to-intermediate distances favor projected and voxel-based completeness, and intermediate distances provide the best trade-off between density, spread, and overall structural representation. In this sense, the fused configuration yields not only more complete geometric coverage, but also a more stable, physically consistent, and information-rich internal distribution of LiDAR returns.

### 4.5. Operating Range, Robustness, and Viewpoint Complementarity

When the geometric, occupancy, and statistical results are considered jointly, the most favorable operating regime for pedestrian representation lies in the short-to-intermediate distance interval, approximately between 3 and 7 m. Within this range, the fused configuration consistently preserves high values of $C_{x}$, $C_{y}$, $C_{z}$, $C_{surf}$, and $S_{3D}$, while still maintaining meaningful voxel occupancy and stable statistical distributions. More specifically, 3 m corresponds to the maximum projected completeness, 2 m to the maximum volumetric occupancy, and 7 m to the maximum global observability score. These results confirm that no single distance simultaneously optimizes all descriptors. Instead, the proposed framework reveals a continuum of operating conditions: 1 m maximizes local sampling density, 2–3 m maximize projected and volumetric completeness, and 7 m provides the best overall balance between geometric extent, projected support, and global observability.

From a practical perspective, however, identifying local maxima is not sufficient for sensor-selection decisions. A sensing configuration should not only perform well at isolated operating points, but should also maintain an acceptable observability level across the full working interval. For this reason, an additional robustness-oriented descriptor was introduced to summarize the global behavior of each sensing configuration over all evaluated distances.

#### 4.5.1. Distance-Robustness Index

To quantify how consistently each sensing configuration preserves observability across the full evaluated range, a Distance-Robustness Index (DRI) was defined from the global observability score $S_{3D}$ as

(14) $${\mathrm{DRI}}_{s}=\frac{1}{N}\sum_{i=1}^{N}S_{3D}^{\left(s\right)}\left(d_{i}\right),$$

where ${\mathrm{DRI}}_{s}$ is the robustness index of sensing configuration *s*, *N* is the total number of evaluated distance conditions, $d_{i}$ denotes the *i*-th distance, and $S_{3D}^{\left(s\right)}\left(d_{i}\right)$ is the corresponding global observability score. In this study, N=12, with $d_{i}\in \left\{1,2,3,4,5,6,7,8,9,10,15,20\right\}$ m.

To complement this mean-performance descriptor, the coefficient of variation (CV) was also computed as

(15) $$CV_{s}=\frac{\sigma_{s}}{\mu_{s}}\times 100,$$

where $\mu_{s}$ and $\sigma_{s}$ are the mean and standard deviation of $S_{3D}$ for sensing configuration *s*, respectively. In this context, the DRI quantifies the average observability level preserved across the full distance range, whereas the CV reflects its relative stability. Lower CV values therefore indicate lower sensitivity to distance variation and, consequently, greater operational consistency.

Using the values reported in Appendix A.2, the resulting indices were ${\mathrm{DRI}}_{TL}=0.4320$, ${\mathrm{DRI}}_{FRL}=0.5040$, and ${\mathrm{DRI}}_{FUSION}=0.5628$. These values show that the fused configuration provides the highest average observability over the complete set of distance conditions. At the same time, it also exhibits the lowest relative variability, with CV=10.7%, indicating that the fused configuration is not only superior on average, but also the most stable across distance. Relative to the best individual sensor, the fused configuration improved the DRI by 11.7% with respect to FRL and by 30.3% with respect to TL. Under this interpretation, the fused TL–FRL configuration should be regarded not merely as the best peak-performing solution, but as the most robust sensing strategy across the entire evaluated operating range.

#### 4.5.2. Complementarity and Overlap Indicator

Although the DRI confirms that fusion is the most robust configuration over distance, it does not by itself explain the origin of this gain. In principle, the advantage of fusion may arise from two different mechanisms: a simple increase in the number of points, or genuine geometric complementarity between the two viewpoints. To distinguish between these effects, an overlap-based complementarity formulation was introduced on the shared canonical occupancy domains [65].

Let $O_{TL}^{\left(p\right)}\left(d\right)$ and $O_{FRL}^{\left(p\right)}\left(d\right)$ denote the occupied sets associated with TL and FRL, respectively, for projection or voxel domain p∈{YZ,XZ,XY,Vox} at distance *d*. The overlap indicator is defined as

(16) $$OI^{\left(p\right)}\left(d\right)=\frac{\left|O_{TL}^{\left(p\right)}\left(d\right)\cap O_{FRL}^{\left(p\right)}\left(d\right)\right|}{\left|O_{TL}^{\left(p\right)}\left(d\right)\cup O_{FRL}^{\left(p\right)}\left(d\right)\right|},$$

and the corresponding complementarity indicator as

(17) $$CI^{\left(p\right)}\left(d\right)=1-OI^{\left(p\right)}\left(d\right).$$

Under this definition, high overlap implies redundancy between viewpoints, whereas high complementarity indicates that the two sensors contribute non-overlapping geometric support to the fused representation.

Because Appendix A.2 reports aggregated occupancy counts rather than raw binary occupancy masks, an exact overlap value cannot be reconstructed directly from the summarized tables. For this reason, a proxy complementarity indicator was computed from the reported occupancy counts as

(18) $$CI_{\mathrm{proxy}}^{\left(p\right)}\left(d\right)=\frac{N_{FUS}^{\left(p\right)}\left(d\right)-\max\left(N_{TL}^{\left(p\right)}\left(d\right),N_{FRL}^{\left(p\right)}\left(d\right)\right)}{N_{FUS}^{\left(p\right)}\left(d\right)},$$

where $N_{TL}^{\left(p\right)}\left(d\right)$, $N_{FRL}^{\left(p\right)}\left(d\right)$, and $N_{FUS}^{\left(p\right)}\left(d\right)$ denote the occupied-cell or occupied-voxel counts of TL, FRL, and the fused configuration, respectively. This quantity measures the fraction of fused support that is not already provided by the best individual sensor.

To summarize the behavior across projected and volumetric domains, a global proxy complementarity index was defined as

(19) $$CI_{\mathrm{proxy},\mathrm{global}}\left(d\right)=\frac{1}{4}\left(CI_{\mathrm{proxy}}^{\left(YZ\right)}\left(d\right)+CI_{\mathrm{proxy}}^{\left(XZ\right)}\left(d\right)+CI_{\mathrm{proxy}}^{\left(XY\right)}\left(d\right)+CI_{\mathrm{proxy}}^{\left(Vox\right)}\left(d\right)\right).$$

Using the values reported in Appendix A.2, the average global proxy complementarity across all evaluated distances was 0.2957, with maximum values at 8 m (0.4130), 7 m (0.4061), and 10 m (0.3956). These results show that the benefit of fusion cannot be explained only by point-count accumulation. Instead, a substantial fraction of the fused support corresponds to genuinely complementary geometric occupancy, particularly in the voxelized volume and in the YZ and XZ projected domains. This interpretation is fully consistent with the viewpoint-dependent behavior identified in the previous subsections: TL better preserves lateral body extent, FRL better preserves vertical structure, and their fusion yields a more complete and more robust target representation because both sensors contribute partially distinct geometric evidence.

Taken together, the operating-range analysis, the DRI, and the complementarity indicator provide a coherent final synthesis of the experimental results. The short-to-intermediate interval between 3 and 7 m emerges as the most favorable regime for balanced pedestrian representation, the fused TL–FRL configuration exhibits the highest average observability and the lowest relative variability across distance, and the observed gain is supported by genuine geometric complementarity rather than by point accumulation alone. Under this interpretation, fusion improves not only the magnitude of the observability descriptors, but also the operational robustness and structural completeness of the pedestrian representation.

#### 4.5.3. Temporal Object-Level Validation via 3D Centroid Sensitivity

To further assess whether the geometric observability gains obtained with the proposed framework are accompanied by a more stable object-level representation, an additional temporal sanity check was conducted using the same ROS 2 bag recordings. For each representative distance, a sequence of 20 consecutive frames was analyzed for the TL, FRL, and FUSION configurations. The goal of this analysis was not to introduce a full detector- or tracker-level benchmark, but rather to verify whether the sensing configurations that provide stronger geometric support also yield a more temporally stable pedestrian representation.

For each sensing configuration s∈{TL,FRL,FUSION} and each valid frame *k*, the local centroid of the pedestrian point cloud was defined as

(20) $$c_{k}^{\left(s\right)}=\left[\begin{array}{c} x_{k}^{\left(s\right)}\\ y_{k}^{\left(s\right)}\\ z_{k}^{\left(s\right)} \end{array}\right].$$

The temporal mean centroid over the $N_{s}$ valid frames was then computed as

(21) $${\bar{c}}^{\left(s\right)}=\frac{1}{N_{s}}\sum_{k=1}^{N_{s}}c_{k}^{\left(s\right)}.$$

Based on this reference, the 3D centroid sensitivity was defined as the Euclidean deviation of each frame centroid from its temporal mean,

(22) $$d_{k}^{\left(s\right)}={∥c_{k}^{\left(s\right)}-{\bar{c}}^{\left(s\right)}∥}_{2}.$$

From the set $\left\{d_{k}^{\left(s\right)}\right\}$, the reported summary statistics were obtained, including the mean sensitivity, standard deviation, minimum and maximum deviation, and the root-mean-square sensitivity

(23) $$d_{\mathrm{RMS}}^{\left(s\right)}=\sqrt{\frac{1}{N_{s}}\sum_{k=1}^{N_{s}}{\left(d_{k}^{\left(s\right)}\right)}^{2}}.$$

Lower values of these descriptors indicate a more compact temporal centroid distribution and, therefore, a more stable object-level representation.

To facilitate interpretation, the empirical centroid sensitivity was also represented through a Gaussian model,

(24) $$p^{\left(s\right)}\left(d\right)=\frac{1}{\sigma_{d}^{\left(s\right)}\sqrt{2\pi }}\exp\;\left(-\frac{{\left(d-\mu_{d}^{\left(s\right)}\right)}^{2}}{2{\left(\sigma_{d}^{\left(s\right)}\right)}^{2}}\right),$$

where $\mu_{d}^{\left(s\right)}$ and $\sigma_{d}^{\left(s\right)}$ denote the mean and standard deviation of the sensitivity distribution for configuration *s*. In this representation, narrower Gaussian profiles correspond to lower centroid dispersion and improved temporal robustness.

The quantitative results are summarized in Table 3. For the representative short- and medium-range cases from 1 to 7 m, the centroid sensitivity remained within the millimeter-to-centimeter scale for all sensing configurations, indicating that the extracted pedestrian representation was temporally stable across the analyzed 20-frame windows. At 1 m, TL achieved the lowest mean sensitivity (0.0066 m), followed by FUSION (0.0072 m) and FRL (0.0075 m). At 3 m, FUSION yielded the lowest mean sensitivity (0.0093 m), slightly improving over FRL (0.0094 m) and more clearly over TL (0.0110 m). At 5 m, FRL produced the lowest mean sensitivity (0.0139 m), while FUSION remained close (0.0145 m) and still improved over TL (0.0161 m). At 7 m, centroid sensitivity decreased substantially for all configurations, with TL, FRL, and FUSION reaching 0.0032 m, 0.0046 m, and 0.0037 m, respectively.

The fused configuration also showed competitive worst-case temporal behavior. In particular, at 7 m it achieved the lowest maximum deviation (0.0130 m), compared with 0.0164 m for TL and 0.0149 m for FRL. This result is especially relevant because 7 m was also identified in the previous subsections as one of the most favorable distances for balanced geometric observability. Therefore, the temporal centroid analysis is consistent with the multimetric observability analysis: the distance regime that provides the best balance of extent, projected support, and global observability also yields a compact and stable object-level centroid distribution.

At 15 m, the temporal behavior becomes more configuration-dependent. In this longer-range condition, FRL provided the most compact centroid distribution, with a mean sensitivity of 0.0105 m and a maximum deviation of 0.0195 m, while FUSION remained intermediate (0.0167 m mean sensitivity), and TL showed the highest temporal dispersion, with a mean sensitivity of 0.0438 m and a maximum deviation of 0.0872 m. This result suggests that, at longer range, the frontal–lateral viewpoint preserves a more stable centroid estimate than the roof-mounted viewpoint, whereas the fused representation still remains substantially more stable than TL alone. Accordingly, the main temporal benefit of fusion is most clearly observed in the representative short-to-intermediate range, where geometric observability was also strongest.

**Figure 13:**
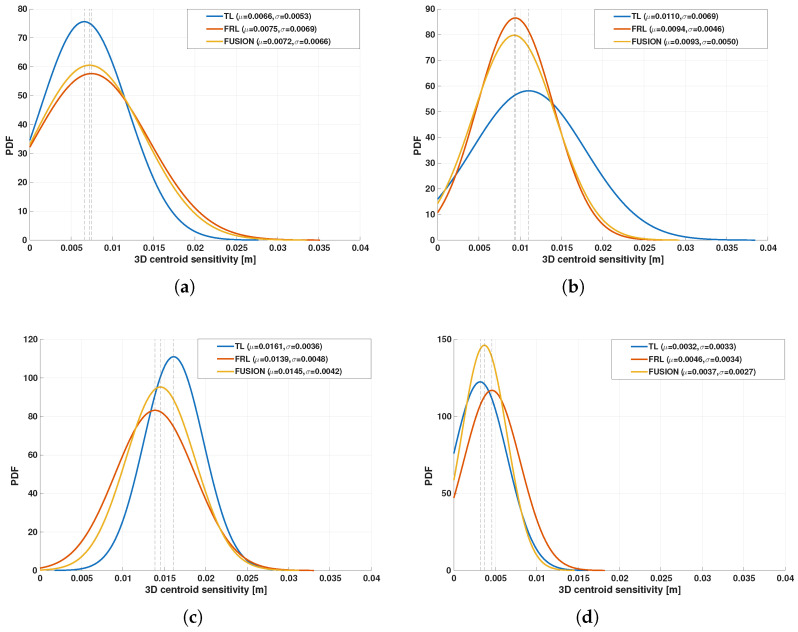
Representative Gaussian centroid-sensitivity profiles at (**a**) 1 m, (**b**) 3 m, (**c**) 5 m, and (**d**) 7 m for TL, FRL, and FUSION. The plots illustrate the temporal dispersion of the global 3D centroid sensitivity and show how the fused configuration maintains a compact centroid distribution across the representative operating range.

Overall, the centroid-sensitivity analysis provides a lightweight object-level validation of the proposed framework. Although it does not replace a full benchmark based on detector accuracy or tracking metrics, it provides direct supporting evidence that the proposed observability framework is not only geometrically meaningful, but also practically relevant for downstream perception tasks. In particular, the results shown in Figure 13 support the interpretation that improved multiview geometric observability is generally accompanied by a temporally consistent pedestrian representation, especially within the operating range where the fused configuration also achieves its best geometric balance.

## 5. Discussion

The results confirm that pedestrian observability is intrinsically viewpoint-dependent, distance-dependent, and irreducibly three-dimensional. Although all evaluated sensing configurations were able to capture the pedestrian target over the analyzed range, the quality of the representation changed substantially with both distance and sensor location. This finding reinforces a central premise of this work: LiDAR-based pedestrian perception should not be interpreted only in terms of point abundance, but rather as the combined effect of geometric extent, projected completeness, volumetric support, and internal spatial distribution of returns. Under this interpretation, observability is better understood as a structured geometric property of the sensed target than as a simple consequence of point count.

A first major outcome is that the distance maximizing observability depends on the descriptor being considered. The shortest evaluated range produced the highest local sampling density and the strongest voxel-density-related statistics, but this dense short-range sampling did not correspond to the highest overall observability. Instead, projected and volumetric completeness were maximized at short-to-intermediate distances, while the highest global observability score $S_{3D}$ was obtained at 7 m for the fused configuration. This distinction is important because it demonstrates that dense local sampling and balanced three-dimensional observability are related but not equivalent. In practical terms, a point cloud may be dense and still fail to preserve a sufficiently complete body representation if its spatial support remains concentrated or anisotropic.

A second key result is the clear complementarity between the two evaluated LiDAR viewpoints. The roof-mounted TL sensor more effectively preserved lateral extent and global body width, which is physically consistent with its elevated perspective and broader top-down view. By contrast, the FRL sensor better preserved vertical structure and scan-layer support, likely due to its lower frontal–lateral perspective and its more favorable intersection with the torso and lower-body regions. These differences confirm that LiDAR mounting position is not a secondary implementation detail, but a design variable that directly conditions which parts of pedestrian geometry are more reliably observed.

The benefit of fusion arises precisely from this complementarity. Across most evaluated distances, the fused configuration achieved higher values than either individual sensor in the main observability indicators, including projected occupancy, volumetric support, and the composite score $S_{3D}$. More importantly, the additional robustness analysis showed that this gain is not restricted to a few isolated distances. The fused configuration yielded the highest Distance-Robustness Index, ${\mathrm{DRI}}_{FUSION}=0.5628$, compared with 0.5040 for FRL and 0.4320 for TL, and it also exhibited the lowest coefficient of variation (CV=10.7%). Therefore, within the evaluated platform and controlled benchmark, fusion should be interpreted not only as the best-performing configuration at the optimal distance, but also as the most stable sensing strategy across the full analyzed operating range.

The overlap-based complementarity analysis further clarifies the mechanism behind this gain. The average global proxy complementarity value of 0.2957 indicates that a substantial fraction of the fused support is not already present in the best individual sensor. The highest complementarity values were observed at 8 m, 7 m, and 10 m, which is especially relevant because this interval overlaps with the range where the fused configuration also showed the strongest balance between geometric extent, projected completeness, and global observability. This means that fusion improves the representation not merely by accumulating more returns, but by integrating partially non-overlapping geometric evidence from two distinct viewpoints. In that sense, the improvement provided by fusion is geometric rather than purely numerical.

The statistical descriptors reinforce this same interpretation. Because the point clouds were analyzed in a pedestrian-centered local frame, the near-zero horizontal centroids confirm that the adopted normalization strategy effectively removed positional bias and allowed the analysis to focus on sensing behavior itself. At 1 m, the Gaussian approximations were narrow and sharply concentrated, indicating dense but spatially localized support. At 3 m, the distributions broadened, especially along the lateral and vertical axes, suggesting improved body support. At 5 m and 7 m, the distributions became more balanced, indicating that body proportions were preserved more consistently even as local point density gradually decreased. These results support the interpretation that the most informative pedestrian representation is not achieved where the cloud is most concentrated, but where density, extent, and spatial spread are jointly balanced.

The additional centroid-based temporal sanity check strengthens the practical interpretation of these findings. Although the proposed framework was not designed as a detector- or tracker-level benchmark, the multi-frame centroid sensitivity analysis, based on 20 consecutive frames for each representative distance, provided an object-level indication of temporal consistency using the same ROS 2 bag data. In the representative short- and medium-range interval from 1 to 7 m, centroid sensitivity remained within the millimeter-to-centimeter scale for all sensing configurations, and the fused configuration consistently remained among the most compact temporal centroid distributions. The lowest maximum deviation was obtained by the fused configuration at 7 m, which is especially meaningful because this distance also corresponded to the best balance of geometric observability. This result supports the interpretation that improved geometric support is accompanied by a temporally stable object-level representation.

At the same time, the temporal results also reveal that the fusion advantage is not uniform under all conditions. At 15 m, FRL produced the most compact centroid distribution, whereas TL exhibited the highest temporal dispersion and the fused configuration remained intermediate. This behavior suggests that, at longer range, the frontal–lateral viewpoint may preserve centroid stability more effectively than the roof-mounted viewpoint for this specific target arrangement. This does not contradict the main findings of the paper; rather, it indicates that the practical benefit of fusion is strongest in the short-to-intermediate operating range where geometric complementarity is better expressed and where the multimetric observability results were also most favorable. Therefore, the present results support a more precise conclusion: fusion is the most robust configuration over the evaluated range as a whole, but its strongest advantages emerge in the representative 3–7 m interval.

From a practical perspective, this interval between approximately 3 and 7 m emerges as the most relevant operating regime for balanced pedestrian representation. Within this range, the fused configuration maintained strong extent ratios together with high projected and volumetric support, while also exhibiting favorable robustness, complementarity, and temporal-stability behavior. This result is particularly meaningful for autonomous driving perception because many interaction-critical pedestrian events occur precisely in near- to mid-range regions where accurate geometric interpretation is essential for detection support, tracking stability, and short-horizon motion reasoning. Although the exact distances associated with local maxima will depend on sensor model, angular resolution, mounting height, and scene conditions, the broader principle suggested by this controlled benchmark is that extremely short distances maximize local density, whereas intermediate distances yield more balanced target observability.

The proposed score $S_{3D}$ also proved useful as an integrative descriptor. Its value lies in combining normalized but complementary observability indicators into a single interpretable quantity that preserves a direct connection to the underlying geometric descriptors. At the same time, the present results show that $S_{3D}$ should not be interpreted in isolation. The individual metrics remain essential because they reveal which specific spatial attributes are preserved or degraded under each sensing condition. The same applies to the DRI, complementarity indicators, and centroid-sensitivity analysis introduced in this work: they do not replace the metric-level analysis, but rather extend it by providing a range-wise robustness summary, a geometric explanation for the fusion gain, and a lightweight object-level check of temporal consistency.

These findings have direct implications for perception-system design. First, they show that LiDAR placement should be treated as an explicit design variable with measurable consequences for pedestrian observability. Second, they suggest that the evaluation of sensing configurations should go beyond raw point counts or detector outputs and include descriptors of geometric completeness, volumetric support, robustness across operating range, viewpoint complementarity, and temporal centroid stability. Third, they support the use of complementary multi-view sensing in compact autonomous platforms where a single LiDAR may under-represent particular body dimensions or projected surfaces. In this sense, the proposed framework can serve as a practical pre-deployment tool for comparing sensor placements and fusion strategies before they are integrated into full perception pipelines.

Several limitations should nevertheless be acknowledged. First, the experiments were conducted under controlled conditions using a single compact autonomous platform, a static pedestrian target, and a canonical reference volume. This was appropriate for isolating the effects of viewpoint and distance, but it does not capture pose changes, articulation, multiple pedestrians, partial occlusion, clutter, or dynamic traffic interactions. Second, the benchmark relied on manually defined ROIs and on a controlled offline analysis, which enabled a clean geometric comparison but may still introduce a degree of selection dependence. Third, the fused configuration was evaluated at the point-cloud level after ROI extraction, which allows a controlled comparison of geometric support but does not yet address synchronization uncertainty, temporal filtering, latency, or the constraints of real-time deployment. Fourth, the Gaussian profiles should be interpreted as parametric summaries of dispersion rather than as empirical distribution models, and the voxel-based descriptors remain dependent on the adopted voxel size and canonical target volume. Finally, the centroid-based temporal validation should be understood as a lightweight object-level sanity check rather than as a substitute for a full benchmark using detector or tracker performance metrics.

These limitations do not reduce the value of the present contribution; rather, they define a clear path for future work. The proposed framework can be extended toward dynamic pedestrian sequences, additional LiDAR mounting positions, temporal fusion across consecutive frames, and benchmarking under occlusion or cluttered urban conditions. It can also be linked explicitly to downstream tasks such as pedestrian detection, tracking, and short-term trajectory prediction in order to examine how improvements in geometric observability translate into task-level perception performance.

Overall, the present discussion supports the main hypothesis of this work: pedestrian observability is not adequately characterized by point density alone, but by the extent to which sensed data preserve a complete, balanced, and structurally meaningful three-dimensional representation of the target. Within the evaluated compact platform and controlled experimental setup, the fused TL–FRL configuration provides the strongest overall observability profile, while the interval between 3 and 7 m emerges as the most favorable regime for balanced pedestrian representation. The proposed framework therefore contributes both a physically grounded interpretation of LiDAR observability and a practical methodology for sensor-placement assessment in autonomous vehicle platforms.

## 6. Conclusions

This work presented a three-dimensional observability framework for evaluating pedestrian representation from complementary LiDAR viewpoints on the ANTA autonomous vehicle platform. By combining geometric extent, projected surface coverage, volumetric occupancy, and statistical descriptors within a canonical pedestrian volume, the proposed methodology provided a more complete characterization of how viewpoint and distance affect LiDAR-based pedestrian representation.

The results showed that pedestrian observability is strongly distance-dependent and cannot be described by a single descriptor. Very short distances maximize local sampling density, whereas short-to-intermediate distances maximize projected and volumetric completeness. The highest global observability score was obtained at 7 m for the fused configuration, indicating that the most informative representation is achieved not at maximum point density, but at the best balance among extent, projected support, volumetric coverage, and spatial distribution.

The comparative analysis also confirmed the complementary roles of the two LiDAR viewpoints. TL more effectively preserved lateral body extent, whereas FRL better captured vertical structure and scan-layer support. Their fusion improved the observability profile across most of the evaluated range by combining distinct geometric perspectives into a more complete three-dimensional representation. This interpretation was reinforced by the robustness and complementarity analyses: the fused configuration achieved the highest Distance-Robustness Index (${\mathrm{DRI}}_{FUSION}=0.5628$) and the lowest coefficient of variation (CV=10.7%), indicating the most stable overall behavior within the evaluated benchmark.

A lightweight temporal object-level validation further supported the practical relevance of the framework. Using 20 consecutive frames per representative distance, the centroid-sensitivity analysis showed that, in the representative 1–7 m range, the pedestrian centroid remained temporally stable for all sensing configurations and that the fused configuration consistently remained among the most compact centroid distributions. At 15 m, however, FRL provided the most stable centroid estimate, indicating that the temporal benefit of fusion is strongest in the short-to-intermediate operating range.

Overall, within the evaluated compact platform and controlled experimental setup, the fused TL–FRL configuration provided the strongest overall observability profile, while the interval between 3 and 7 m emerged as the most favorable operating region for balanced pedestrian representation. Future work will extend this framework toward dynamic pedestrian scenarios, additional sensor placements, temporal fusion, and stronger links to downstream tasks such as detection, tracking, and short-term motion prediction.

## Figures and Tables

**Table 1:** Comparative LiDAR-based pedestrian perception approaches.

Ref.	Datasets	Algorithms	SensorDesign	Geometric Perception	EvaluationMetrics	Research Line
[19,20,21,22]	Public/ Experimental	Multimodal CNN-based fusion	Multimodal sensor fusion	Implicit geometry	mAP, IoU, Precision/Recall	Multimodal fusion
[23,24,25]	Public	CNN-based (VoxelNet, PV-RCNN)	Single LiDAR (fixed)	None (detection-driven)	mAP, IoU, AP_3*D*_	Algorithm-driven detection
[26,27,28,29]	Public/ Simulated	Reconstruction (DL-based)	Simulation-based modeling	Reconstruction-based	Chamfer Dist., IoU, RMSE	Geometry Reconstruction
[30,31]	Public/ Experimental	CNN + meta-learning	Single LiDAR (fixed)	Implicit geometry	mAP, Recall	Occlusion-aware detection
[21,22,32,33,34,35,36,37]	Experimental/ Simulated	Fusion + physical models	Multimodal fusion	Implicit geometry	mAP, Detection rate	Adverse-condition robustness
[38,39,40,41,42]	Experimental/ Simulated	Model-based	Hardware-level optimization	Projection-based (limited)	Coverage, FOV, resolution	Hardware and sensor design
[43,44,45,46,47,48,49,50,51]	Public	DL benchmarking	No sensor analysis	None	mAP, ADE, FDE	Evaluation benchmarking
Ours	Experimental	LiDAR Fusion	Multi-View LiDAR Fusion	Projection-based, Volumetric, and Statistical Descriptors	Voxels, Volumetric, $CV_{s}$, $DRI_{s}$, OI, CI	Geometry-driven observability and Sensor Configuration

**Table 2:** Best-performing configurations for each observability metric, indicating the sensor and distance at which the maximum value is achieved.

Metric	Sensor	Distance (m)	Best Value
Cx	FUSION	7	0.9690
Cy	TL	6	0.9602
Cz	FRL	9	0.9482
Cyz	FUSION	3	0.3677
Cxz	FUSION	3	0.3039
Cxy	FUSION	7	0.7083
Csurf	FUSION	3	0.4334
Cvol	FUSION	2	0.0948
NumPtsInside	FUSION	1	1513
NumOccYZ	FUSION	3	150
NumOccXZ	FUSION	3	62
NumOccXY	FUSION	7	51
NumOccVox	FUSION	2	232
NumLayersDetected	FRL	2	22
Score3D	FUSION	7	0.6235

**Table 3:** Results of 3D sensitivity and centroid deviation grouped by distance and sensor.

Distance	Sensor	MeanSens.	StdSens.	MinSens.	MaxSens.	RMSSens.	MeanC.X	MeanC.Y	MeanC.Z	MaxDev.
1	TL	0.0066	0.0053	0.0025	0.0224	0.0084	0.0131	0.0182	1.5069	0.0224
	FRL	0.0075	0.0069	0.0014	0.0294	0.0101	0.0037	−0.0665	0.9845	0.0294
	FUSION	0.0072	0.0066	0.0012	0.0279	0.0097	0.0042	−0.0620	1.0124	0.0279
3	TL	0.0110	0.0069	0.0030	0.0326	0.0129	−0.0414	−0.0183	1.0520	0.0326
	FRL	0.0094	0.0046	0.0029	0.0207	0.0104	−0.0112	−0.0046	0.8927	0.0207
	FUSION	0.0093	0.0050	0.0016	0.0238	0.0105	−0.0213	−0.0092	0.9459	0.0238
5	TL	0.0161	0.0036	0.0100	0.0234	0.0165	0.0050	0.0334	0.6827	0.0234
	FRL	0.0139	0.0048	0.0041	0.0254	0.0147	0.0420	0.0478	0.8655	0.0254
	FUSION	0.0145	0.0042	0.0072	0.0242	0.0151	0.0253	0.0413	0.7829	0.0242
7	TL	0.0032	0.0033	0.0011	0.0164	0.0045	−0.0328	0.0258	0.7003	0.0164
	FRL	0.0046	0.0034	0.0008	0.0149	0.0057	−0.0053	0.0471	0.8751	0.0149
	FUSION	0.0037	0.0027	0.0014	0.0130	0.0045	−0.0179	0.0374	0.7953	0.0130
15	TL	0.0438	0.0210	0.0059	0.0872	0.0483	−0.1045	−0.0010	0.6157	0.0872
	FRL	0.0105	0.0044	0.0023	0.0195	0.0114	−0.0849	0.0788	0.9547	0.0195
	FUSION	0.0167	0.0080	0.0016	0.0319	0.0185	−0.0905	0.0565	0.8590	0.0319

Values are grouped by distance and sensing configuration and are reported with four decimal places to preserve the resolution of the temporal sensitivity analysis.

**Figure A1:**
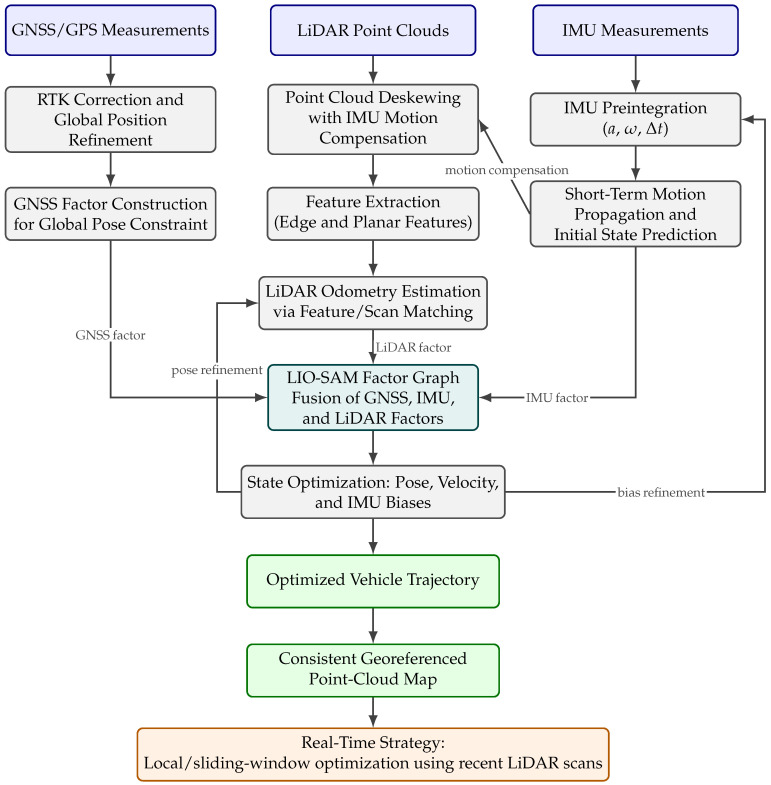
Detailed workflow of the LIO-SAM-based multimodal mapping framework used in ANTA. RTK-corrected GNSS/GPS measurements, preintegrated IMU data, and LiDAR-derived geometric features are fused through factor-graph optimization to estimate pose, velocity, and IMU biases, thereby enabling the generation of a consistent georeferenced point-cloud map for autonomous navigation.

## Data Availability

The Multi-View LiDAR Dataset for Pedestrian Observability in Autonomous Driving is publicly available through IEEE DataPort (https://dx.doi.org/10.21227/86w1-v639, accessed on 25 March 2026), enabling reproducibility and further research on multi-view LiDAR-based pedestrian observability.

## Abbreviations

The following abbreviations are used in this manuscript:

3D	Three-Dimensional
ANTA	Autonomous vehicle prototype developed at Universidad Politécnica Salesiana
ADE	Average Displacement Error
AP3D	Average Precision in 3D Space
CI	Complementarity Indicator
CNN	Convolutional Neural Network
CV	Coefficient of Variation
DL	Deep Learning
DRI	Distance-Robustness Index
FDE	Final Displacement Error
FOV	Field of View
FPS	Frames Per Second
FRL	Front-Right LiDAR
GNSS	Global Navigation Satellite System
GPS	Global Positioning System
IMU	Inertial Measurement Unit
IoU	Intersection over Union
LiDAR	Light Detection and Ranging
LIO-SAM	LiDAR-Inertial Odometry via Smoothing and Mapping
mAP	mean Average Precision
OI	Overlap Indicator
PDF	Probability Density Function
RMSE	Root Mean Square Error
ROS 2	Robot Operating System 2
ROI	Region of Interest
RTK	Real-Time Kinematic
TL	Top LiDAR
$S_{3D}$	Global Three-Dimensional Observability Score

## Appendix A

### Appendix A.1. Georeferenced Mapping for Experimental Deployment

To obtain these representations, the proposed framework employed LIO-SAM to fuse LiDAR, inertial, and GNSS measurements within a tightly coupled localization and mapping pipeline. GNSS/GPS observations were first refined through RTK correction to improve global positioning accuracy and to introduce absolute constraints into the optimization process. In parallel, IMU measurements were preintegrated to estimate short-term motion, propagate the system state, and support LiDAR motion compensation. Based on this inertial prediction, LiDAR point clouds were deskewed and processed to extract discriminative geometric features, which were subsequently used for LiDAR odometry estimation.

The resulting LiDAR odometry was then integrated with IMU and GNSS factors within the LIO-SAM factor graph, where pose, velocity, and IMU biases were jointly optimized. The optimized trajectory enabled the construction of a consistent georeferenced point-cloud map, which served as the geometric basis for high-definition map generation. In a subsequent stage, TIER IV tools were used to create a Lanelet-based road map describing the drivable topology and semantic structure of the environment. This map was then deployed in Autoware Universe for localization, route planning, behavior planning, and vehicle control.

To preserve real-time capability, the implementation emphasized local optimization over recent LiDAR scans using a sliding-window strategy. In this way, the mapping workflow integrates multimodal sensing, LiDAR–inertial odometry, georeferenced reconstruction, and semantic road modeling into a unified framework for autonomous-vehicle deployment. Figure A1 summarizes the LIO-SAM-based mapping workflow adopted in ANTA [66].

### Appendix A.2.

This appendix compiles the complete numerical results supporting the analyses presented in the main text. The tables are organized according to the main groups of descriptors introduced in Section 3, namely geometric extent, occupancy-related observability metrics, local positional statistics, and voxel-based statistical descriptors. These supplementary data are included to improve transparency, facilitate reproducibility, and provide detailed numerical support for the trends discussed in Section 4 and Section 5. For compactness, some table headers are abbreviated; their definitions follow the notation introduced in the methodological section.

**Table A1:** Geometric extent, projected surface coverage, and volumetric occupancy metrics grouped by distance and sensing configuration.

Dist.	Sensor	NumPts	Cx	Cy	Cz	Cyz	Cxz	Cxy	Csurf	Cvol
1	TL	84	0.4655	0.2803	0.0610	0.0221	0.0392	0.1806	0.0660	0.0078
	FRL	1409	0.6029	0.8003	0.3293	0.2255	0.1814	0.4028	0.2588	0.0572
	FUSION	1513	0.6029	0.8149	0.4823	0.2672	0.2157	0.4306	0.2951	0.0682
2	TL	188	0.5521	0.7727	0.2620	0.0931	0.0833	0.4444	0.1785	0.0241
	FRL	776	0.5913	0.8884	0.6001	0.3407	0.2647	0.4722	0.3546	0.0837
	FUSION	965	0.6181	0.9027	0.5901	0.3627	0.2941	0.5694	0.3973	0.0948
3	TL	222	0.7843	0.8843	0.4360	0.1593	0.1127	0.6111	0.2606	0.0351
	FRL	456	0.7264	0.8247	0.8035	0.2819	0.2500	0.5278	0.3354	0.0682
	FUSION	678	0.8674	0.9202	0.7968	0.3676	0.3039	0.6944	0.4334	0.0931
4	TL	168	0.6072	0.8734	0.6435	0.1520	0.0980	0.5417	0.2359	0.0335
	FRL	296	0.7146	0.8534	0.8895	0.2279	0.2108	0.5278	0.2986	0.0568
	FUSION	465	0.6689	0.8792	0.8089	0.3039	0.2549	0.6528	0.3789	0.0784
5	TL	141	0.6628	0.9310	0.7448	0.1691	0.1275	0.5000	0.2414	0.0384
	FRL	172	0.7379	0.8589	0.8833	0.1691	0.1373	0.5417	0.2543	0.0413
	FUSION	313	0.8040	0.9310	0.8873	0.3162	0.2549	0.6528	0.3850	0.0715
6	TL	88	0.6880	0.9602	0.5757	0.1152	0.0882	0.4861	0.2012	0.0245
	FRL	115	0.7067	0.9103	0.9122	0.1422	0.1225	0.5556	0.2406	0.0327
	FUSION	202	0.8327	0.9567	0.8624	0.2426	0.2157	0.6667	0.3419	0.0535
7	TL	73	0.7174	0.9536	0.6538	0.1078	0.0686	0.5000	0.1961	0.0233
	FRL	88	0.7538	0.9057	0.7438	0.1152	0.0931	0.4583	0.1955	0.0245
	FUSION	160	0.9690	0.9280	0.8752	0.2132	0.1569	0.7083	0.3229	0.0458
8	TL	66	0.5916	0.9403	0.7432	0.1103	0.0735	0.4306	0.1812	0.0208
	FRL	68	0.5668	0.8690	0.8580	0.1127	0.0833	0.4028	0.1779	0.0221
	FUSION	134	0.6643	0.9421	0.9306	0.2010	0.1569	0.5972	0.2890	0.0413
9	TL	48	0.7158	0.9222	0.6099	0.0858	0.0735	0.3750	0.1550	0.0172
	FRL	64	0.7092	0.8665	0.9482	0.1127	0.0735	0.4722	0.1928	0.0221
	FUSION	111	0.8225	0.9250	0.9466	0.1961	0.1373	0.5556	0.2712	0.0372
10	TL	41	0.5822	0.8821	0.6771	0.0858	0.0637	0.4167	0.1630	0.0159
	FRL	41	0.5127	0.7790	0.6425	0.0735	0.0441	0.2917	0.1207	0.0131
	FUSION	82	0.7919	0.8821	0.8304	0.1544	0.1127	0.5694	0.2478	0.0282
15	TL	30	0.5380	0.8967	0.4929	0.0613	0.0441	0.3056	0.1181	0.0114
	FRL	38	0.6549	0.7311	0.7086	0.0760	0.0735	0.3194	0.1362	0.0139
	FUSION	67	0.7330	0.8967	0.7201	0.0931	0.0735	0.5000	0.1900	0.0225
20	TL	19	0.4128	0.7518	0.5326	0.0441	0.0441	0.2083	0.0852	0.0078
	FRL	27	0.5342	0.6139	0.7657	0.0588	0.0588	0.2778	0.1136	0.0102
	FUSION	45	0.5366	0.7928	0.8251	0.1005	0.0882	0.3611	0.1626	0.0172

Values are reported for each evaluated distance and sensing configuration (TL, FRL, and FUSION).

**Table A2:** Occupancy-related descriptors, detected scan layers, and global 3D observability score grouped by distance and sensing configuration.

Dist.	Sensor	NumPts	OccYZ	OccXZ	OccXY	OccVox	Layers	Score3D
1	TL	84	9	8	13	19	4	0.1559
	FRL	1409	92	37	29	140	16	0.3960
	FUSION	1513	109	44	31	167	20	0.4467
2	TL	188	38	17	32	59	9	0.3434
	FRL	776	139	54	34	205	22	0.5041
	FUSION	965	148	60	41	232	21	0.5192
3	TL	222	65	23	44	86	12	0.4627
	FRL	456	115	51	38	167	18	0.5555
	FUSION	678	150	62	50	228	20	0.6187
4	TL	168	62	20	39	82	11	0.4805
	FRL	296	93	43	38	139	16	0.5713
	FUSION	465	124	52	47	192	17	0.5699
5	TL	141	69	26	36	94	12	0.5278
	FRL	172	69	28	39	101	10	0.5624
	FUSION	313	129	52	47	175	15	0.6199
6	TL	88	47	18	35	60	5	0.4843
	FRL	115	58	25	40	80	9	0.5708
	FUSION	202	99	44	48	131	10	0.6109
7	TL	73	44	14	36	57	5	0.5057
	FRL	88	47	19	33	60	8	0.5242
	FUSION	160	87	32	51	112	10	0.6235
8	TL	66	45	15	31	51	5	0.5030
	FRL	68	46	17	29	54	7	0.5133
	FUSION	134	82	32	43	101	9	0.5868
9	TL	48	35	15	27	42	4	0.4787
	FRL	64	46	15	34	54	6	0.5597
	FUSION	111	80	28	40	91	10	0.6067
10	TL	41	35	13	30	39	4	0.4688
	FRL	41	30	9	21	32	4	0.4201
	FUSION	82	63	23	41	69	8	0.5580
15	TL	30	25	9	22	28	3	0.4092
	FRL	38	31	15	23	34	4	0.4516
	FUSION	67	38	15	36	55	4	0.5118
20	TL	19	18	9	15	19	3	0.3640
	FRL	27	24	12	20	25	4	0.4191
	FUSION	45	41	18	26	42	6	0.4813

Values are grouped by evaluated distance and sensing configuration.

**Table A3:** Local positional statistics (mean, variance, and standard deviation) grouped by distance and sensing configuration.

Dist.	Sensor	MeanX	MeanY	MeanZ	VarX	VarY	VarZ	StdX	StdY	StdZ
1	TL	0.0129	0.0940	1.5074	0.0013	0.0020	0.0013	0.0356	0.0446	0.0361
	FRL	0.0029	0.0000	0.9915	0.0014	0.0121	0.0236	0.0373	0.1098	0.1535
	FUSION	0.0035	0.0052	1.0205	0.0014	0.0120	0.0364	0.0373	0.1094	0.1908
2	TL	0.0125	0.0238	1.2602	0.0017	0.0123	0.0178	0.0409	0.1108	0.1334
	FRL	0.0011	0.0022	0.9414	0.0012	0.0163	0.0701	0.0344	0.1276	0.2647
	FUSION	0.0033	0.0064	1.0035	0.0013	0.0156	0.0758	0.0360	0.1248	0.2754
3	TL	−0.0151	−0.0019	1.0480	0.0027	0.0191	0.0471	0.0515	0.1381	0.2170
	FRL	0.0134	0.0117	0.8982	0.0014	0.0147	0.1364	0.0378	0.1210	0.3693
	FUSION	0.0040	0.0072	0.9473	0.0020	0.0161	0.1121	0.0448	0.1270	0.3347
4	TL	−0.0164	−0.0051	0.8416	0.0022	0.0178	0.0974	0.0465	0.1336	0.3121
	FRL	0.0158	0.0069	0.8032	0.0018	0.0152	0.1608	0.0422	0.1233	0.4010
	FUSION	0.0042	0.0026	0.8171	0.0022	0.0162	0.1382	0.0465	0.1272	0.3717
5	TL	−0.0202	−0.0029	0.6876	0.0019	0.0202	0.1318	0.0434	0.1420	0.3631
	FRL	0.0180	0.0048	0.8582	0.0023	0.0153	0.1837	0.0481	0.1238	0.4286
	FUSION	0.0008	0.0014	0.7815	0.0025	0.0175	0.1675	0.0498	0.1323	0.4093
6	TL	−0.0189	−0.0060	0.6650	0.0016	0.0208	0.0869	0.0397	0.1444	0.2948
	FRL	0.0313	0.0140	0.8824	0.0026	0.0169	0.1575	0.0506	0.1301	0.3969
	FUSION	0.0096	0.0053	0.7881	0.0028	0.0187	0.1385	0.0525	0.1368	0.3721
7	TL	−0.0024	−0.0083	0.6742	0.0026	0.0201	0.0800	0.0512	0.1418	0.2828
	FRL	0.0215	0.0058	0.8395	0.0042	0.0165	0.1532	0.0645	0.1284	0.3914
	FUSION	0.0113	−0.0002	0.7691	0.0036	0.0181	0.1287	0.0604	0.1344	0.3587
8	TL	−0.0125	−0.0192	0.6018	0.0013	0.0211	0.1384	0.0364	0.1451	0.3720
	FRL	0.0162	0.0151	0.9118	0.0017	0.0162	0.1805	0.0416	0.1275	0.4249
	FUSION	0.0022	−0.0017	0.7601	0.0017	0.0189	0.1839	0.0417	0.1375	0.4289
9	TL	−0.0041	−0.0165	0.6270	0.0018	0.0198	0.1137	0.0422	0.1406	0.3372
	FRL	0.0003	0.0203	0.8414	0.0024	0.0167	0.2587	0.0486	0.1294	0.5086
	FUSION	−0.0016	0.0046	0.7498	0.0021	0.0184	0.2080	0.0460	0.1355	0.4561
10	TL	−0.0190	−0.0152	0.5306	0.0020	0.0195	0.1273	0.0448	0.1395	0.3568
	FRL	0.0185	0.0229	0.9646	0.0016	0.0155	0.1328	0.0395	0.1244	0.3644
	FUSION	−0.0002	0.0038	0.7476	0.0021	0.0178	0.1772	0.0462	0.1335	0.4209
15	TL	−0.0199	−0.0338	0.5595	0.0021	0.0228	0.0891	0.0462	0.1509	0.2986
	FRL	0.0257	0.0270	0.9655	0.0021	0.0151	0.1689	0.0462	0.1228	0.4110
	FUSION	0.0051	−0.0004	0.7825	0.0026	0.0195	0.1737	0.0515	0.1395	0.4168
20	TL	−0.0300	−0.0330	0.5156	0.0015	0.0163	0.1073	0.0381	0.1277	0.3275
	FRL	0.0038	0.0317	1.0341	0.0015	0.0109	0.1881	0.0388	0.1043	0.4337
	FUSION	−0.0106	0.0041	0.8134	0.0018	0.0142	0.2194	0.0420	0.1192	0.4684

Values are grouped by evaluated distance and sensing configuration.

**Table A4:** Occupancy-ratio and voxel-based statistical descriptors grouped by distance and sensing configuration.

Dist.	Sensor	OccR.YZ	OccR.XZ	OccR.XY	OccR.Vox	MeanV.A.	VarV.A.	StdV.A.	MeanV.O.	VarV.O.	StdV.O.
1	TL	0.0294	0.0441	0.1389	0.0082	0.0359	0.2463	0.4962	4.4000	10.9400	3.3076
	FRL	0.2475	0.1961	0.4028	0.0629	0.6046	8.7358	2.9556	9.6104	52.3157	7.2330
	FUSION	0.2770	0.2402	0.4306	0.0711	0.6405	8.9386	2.9897	9.0115	50.3217	7.0938
2	TL	0.1054	0.0931	0.4583	0.0245	0.0809	0.3464	0.5886	3.3000	3.5100	1.8735
	FRL	0.3382	0.2843	0.4861	0.0817	0.3342	1.7421	1.3199	4.0900	5.9619	2.4417
	FUSION	0.3848	0.3137	0.5694	0.0993	0.4150	2.1701	1.4731	4.1811	6.1154	2.4729
3	TL	0.1716	0.1324	0.6250	0.0404	0.0956	0.2825	0.5315	2.3636	1.6253	1.2749
	FRL	0.3039	0.2892	0.5417	0.0694	0.1961	0.6691	0.8180	2.8235	2.2159	1.4886
	FUSION	0.3922	0.3431	0.6944	0.1005	0.2917	1.0269	1.0133	2.9024	2.6409	1.6251
4	TL	0.1593	0.1225	0.5972	0.0368	0.0723	0.1667	0.4083	1.9667	0.8100	0.9000
	FRL	0.2426	0.2108	0.5694	0.0568	0.1275	0.3498	0.5914	2.2446	1.4078	1.1865
	FUSION	0.3652	0.2892	0.6944	0.0907	0.1998	0.5283	0.7269	2.2027	1.4139	1.1891
5	TL	0.1789	0.1275	0.5139	0.0380	0.0605	0.1115	0.3340	1.5914	0.4997	0.7069
	FRL	0.1789	0.1520	0.5278	0.0408	0.0739	0.1518	0.3896	1.8100	0.5739	0.7576
	FUSION	0.3137	0.2500	0.6667	0.0764	0.1344	0.2626	0.5124	1.7594	0.5785	0.7606
6	TL	0.1152	0.0882	0.5000	0.0257	0.0376	0.0599	0.2447	1.4603	0.2484	0.4984
	FRL	0.1397	0.1324	0.5139	0.0306	0.0490	0.0883	0.2971	1.6000	0.4000	0.6325
	FUSION	0.2549	0.2206	0.6944	0.0564	0.0866	0.1445	0.3801	1.5362	0.3356	0.5793
7	TL	0.1005	0.0686	0.4583	0.0208	0.0282	0.0421	0.2052	1.3529	0.2284	0.4779
	FRL	0.1225	0.0931	0.5278	0.0249	0.0380	0.0627	0.2504	1.5246	0.2494	0.4994
	FUSION	0.2083	0.1569	0.7083	0.0458	0.0662	0.1026	0.3204	1.4464	0.2471	0.4971
8	TL	0.1103	0.0784	0.4444	0.0221	0.0282	0.0396	0.1991	1.2778	0.2006	0.4479
	FRL	0.1103	0.0931	0.4306	0.0221	0.0294	0.0433	0.2080	1.3333	0.2222	0.4714
	FUSION	0.2132	0.1618	0.5972	0.0433	0.0576	0.0861	0.2935	1.3302	0.2966	0.5446
9	TL	0.0907	0.0637	0.3750	0.0172	0.0204	0.0265	0.1629	1.1905	0.1542	0.3927
	FRL	0.1078	0.0686	0.4444	0.0212	0.0274	0.0389	0.1972	1.2885	0.2053	0.4530
	FUSION	0.1985	0.1324	0.5694	0.0384	0.0478	0.0643	0.2536	1.2447	0.1848	0.4299
10	TL	0.0882	0.0588	0.4028	0.0155	0.0176	0.0213	0.1461	1.1316	0.1143	0.3380
	FRL	0.0809	0.0588	0.3472	0.0143	0.0176	0.0238	0.1542	1.2286	0.1763	0.4199
	FUSION	0.1691	0.1176	0.5694	0.0298	0.0351	0.0445	0.2110	1.1781	0.1464	0.3826
15	TL	0.0637	0.0441	0.3056	0.0106	0.0131	0.0178	0.1334	1.2308	0.1775	0.4213
	FRL	0.0809	0.0588	0.3611	0.0143	0.0159	0.0189	0.1376	1.1143	0.1012	0.3182
	FUSION	0.1005	0.0784	0.5139	0.0241	0.0290	0.0388	0.1969	1.2034	0.1959	0.4426
20	TL	0.0441	0.0343	0.2222	0.0074	0.0082	0.0097	0.0987	1.1111	0.0988	0.3143
	FRL	0.0613	0.0539	0.2500	0.0106	0.0110	0.0117	0.1083	1.0385	0.0370	0.1923
	FUSION	0.1054	0.0882	0.3611	0.0180	0.0192	0.0213	0.1459	1.0682	0.0635	0.2521

Values are grouped by evaluated distance and sensing configuration.

**Table A5:** Proxy complementarity indicator computed from the Appendix A occupancy counts.

Distance (m)	${\mathrm{CI}}_{\mathrm{proxy}}^{\mathrm{YZ}}$	${\mathrm{CI}}_{\mathrm{proxy}}^{\mathrm{XZ}}$	${\mathrm{CI}}_{\mathrm{proxy}}^{\mathrm{XY}}$	${\mathrm{CI}}_{\mathrm{proxy}}^{\mathrm{Vox}}$	${\mathrm{CI}}_{\mathrm{proxy},\mathrm{global}}$
1	0.1560	0.1591	0.0645	0.1617	0.1353
2	0.0608	0.1000	0.1707	0.1164	0.1120
3	0.2333	0.1774	0.1200	0.2675	0.1996
4	0.2500	0.1731	0.1702	0.2760	0.2173
5	0.4651	0.4615	0.1702	0.4229	0.3799
6	0.4141	0.4318	0.1667	0.3893	0.3505
7	0.4598	0.4062	0.2941	0.4643	0.4061
8	0.4390	0.4688	0.2791	0.4653	0.4130
9	0.4250	0.4643	0.1500	0.4066	0.3615
10	0.4444	0.4348	0.2683	0.4348	0.3956
15	0.1842	0.0000	0.3611	0.3818	0.2318
20	0.4146	0.3333	0.2308	0.4048	0.3459
